# A Reappraisal of Dependency Length Minimization as a Linguistic Universal

**DOI:** 10.1162/opmi_a_00060

**Published:** 2022-09-15

**Authors:** Himanshu Yadav, Shubham Mittal, Samar Husain

**Affiliations:** Department of Linguistics, University of Potsdam, Germany; Department of Chemical Engineering, Indian Institute of Technology Delhi, India; Department of Humanities and Social Sciences, Indian Institute of Technology Delhi, India

**Keywords:** dependency length minimization, syntactic complexity, working-memory constraints

## Abstract

Dependency length minimization is widely regarded as a cross-linguistic universal reflecting syntactic complexity in natural languages. A typical way to operationalize dependency length in corpus-based studies has been to count the number of words between syntactically related words. However, such a formulation ignores the *syntactic nature* of the linguistic material that intervenes a dependency. In this work, we investigate if the number of syntactic heads (rather than the number of words) that intervene a dependency better captures the syntactic complexity across languages. We demonstrate that the dependency length minimization constraint in terms of the number of words could arise as a consequence of constraints on the intervening heads and the tree properties such as node arity. The current study highlights the importance of syntactic heads as central regions of structure building during processing. The results show that when syntactically related words are nonadjacent, increased structure building in the intervening region is avoided.

## INTRODUCTION

Natural languages have been argued to be shaped by communicative pressures as well as certain cognitive constraints such as limited working memory (Bickerton, 2003; Hawkins, 2014; Hockett, 1960; Jaeger & Tily, 2011; Zipf, 1949). Such accounts contend that efficiency in formulating and comprehending a language dictates its formal properties (Bybee, 2006; Croft, 2001; Gibson et al., 2019; Haspelmath, 2008; Hawkins, 1994; Piantadosi et al., 2012) and is a vital determinant of a language’s communicative utility. In the sentence processing literature, a dominant way to operationalize and test this efficiency has been in terms of the linear arrangement of syntactically related words (e.g., a verb and its nominal arguments) (Futrell et al., 2020). The hypothesis, termed *dependency length minimization* (DLM), holds that, on average, the distance between a head (e.g., a verb) and its dependent (e.g., a noun) is minimized in natural languages (Behagel, 1930; Gibson, 1998; Gildea & Temperley, 2007; Hawkins, 1990, 2014; Hudson, 1995; Rijkhoff, 1986; Temperley & Gildea, 2018). Why should dependencies be short? Theories of sentence processing maintain that syntactic dependencies (e.g., the syntactic relation between the verb “ate” and “John”/“a mango” in *John ate a mango*) need to be established in order to comprehend or produce a sentence. Dependency resolution between a pair of words typically involves one of the words to be temporarily retained in memory. Under the assumption of limited working memory (Baddeley & Hitch, 1974; Cowan, 2001; Miller, 1956; see Miyake & Shah, 1999, for an extensive overview), longer dependencies could lead to retrieval failure due to decay or interference-driven constraints (Bartek et al., 2011; Grodner & Gibson, 2005; Lewis & Vasishth, 2005). Indeed, longer syntactic dependencies have been shown to pose more difficulty during both comprehension and generation (Bartek et al., 2011; Grodner & Gibson, 2005; Scontras et al., 2017). Recent large-scale cross-linguistic corpus investigations have provided a strong validation for the DLM hypothesis (Futrell et al., 2015; Liu, 2008; Liu et al., 2017). Based on this line of research, DLM has been claimed to be a linguistic universal showcasing the influence of communicative pressure and cognitive constraints on language forms (Futrell et al., 2020). For example, it has been argued to determine some critical properties of languages, such as, the rarity of discontiguous phrases (Ferrer-i Cancho, 2006). Relatedly, it has been argued that the occurrence of the two most frequent word orders (Subject-Verb-Object, and Subject-Object-Verb) across languages can be explained by such minimization pressures during comprehension (Hawkins, 1990).

Dependency length in large-scale corpus studies (e.g., Futrell et al., 2015) has typically been operationalized by counting the number of words between syntactically related words. However, in the larger literature, dependency length has been computed using a variety of ways, for example, number of discourse referents (Gibson, 1998), number of phrasal nodes (Ferreira, 1991), number of words (Temperley, 2007), and so on. Previous studies comparing the effectiveness of such metrics have argued that these metrics (e.g., counting number of words vs. counting number of phrases) are largely interchangeable (Szmrecsányi, 2004; Wasow, 1997). This would suggest that computing dependency length using any of these measures should be equally effective in capturing linguistic complexity. However, a large-scale corpus study that tests the possible interaction or independence of various metrics is currently lacking.

**Figure 1.**
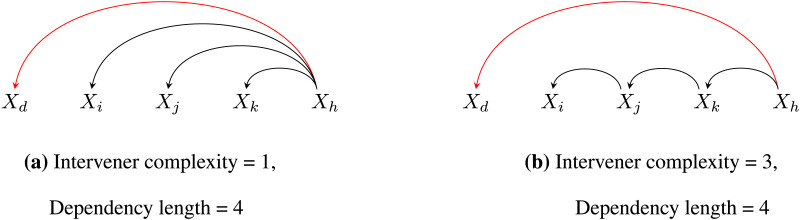
**Dependency structures with varying intervener complexity for *X*_*h*_ → *X*_*d*_.** While the dependency lengths (number of words that intervene *X*_*h*_ → *X*_*d*_) in tree (a) and tree (b) are the same, the two structures differ in their intervener complexity (the number of intervening heads).

Operationalizing dependency length in terms of the number of words ignores the *syntactic nature* of the linguistic material that intervenes a dependency. Given the limited memory resource, it is reasonable to assume that more structure building in the intervening region should lead to more difficultly in processing the unresolved dependency. Consistent with this idea, there is evidence that not only the number but the complexity of the words that intervene a syntactic dependency matters (e.g., Gibson & Thomas, 1999; Wasow & Arnold, 2003; Yadav et al., 2020). For example, it has been shown that introducing clausal embeddings can lead to forgetting effects during comprehension (Gibson & Thomas, 1999). Similarly, Wasow and Arnold (2003) found an independent effect of phrasal complexity on noun phrase shifts and dative alternations. Interestingly, while Wasow and Arnold (2003) argue for an independent effect of both length and phrasal complexity, others have proposed that phrasal length is not an appropriate metric to quantify syntactic complexity (Chomsky, 1975). This line of work predicts that the complexity of the linguistic material that intervenes a syntactic dependency will be minimized. We call this the intervener complexity minimization (ICM) hypothesis. In this work, we operationalized complexity as the number of syntactic heads that intervene a dependency (Yadav et al., 2017, 2020; see Figure 1).

The rationale behind using the number of intervening heads as a measure of complexity comes from the proposal that both structural integrations and temporary storage of linguistic items consume the same pool of *limited resources* (Gibson, 1998; Just & Carpenter, 1992). For example, in Figure 1, the node *X*_*d*_ has to be actively maintained in memory until the comprehender resolves the dependency *X*_*h*_ → *X*_*d*_. In Figure 1(b), compared to Figure 1(a), more numbers of structural integrations are required in the region intervening *X*_*h*_ and *X*_*d*_, that is, *X*_*j*_ → *X*_*i*_, *X*_*k*_ → *X*_*j*_ need to be resolved. Since these integrations are assumed to consume the same pool of limited resources, the maintenance of node *X*_*d*_ should become more difficult in Figure 1(b) compared to Figure 1(a), and hence cause more difficulty in resolving the *X*_*h*_ → *X*_*d*_ dependency in Figure 1(b) than in Figure 1(a). In sum, the number of intervening heads represents the amount of resource demand due to structural integrations in the intervening region of a dependency.^1^ The ICM hypothesis states that the intervener complexity, that is, the number of heads intervening a dependency, is minimized in natural languages. The DLM hypothesis based on the number of words does not make any prediction regarding the nature of words that intervene a dependency.

While the ICM hypothesis tests if intervener complexity (IC) is minimized in natural language, it does not test how IC and dependency length (DL) interact. Recall that previous work (Wasow & Arnold, 2003) suggests that both have independent influence on the complexity of a sentence. Given that the dependency length is an upper bound on the intervener complexity there are two ways in which DL and IC could interact in capturing syntactic complexity across languages. The first possibility is that a constraint on IC and a constraint on DL independently shape the pattern of linguistic structures. One can ask whether the intervener complexity is minimized *independent* of the minimization of dependency length. We term this as the ICM as an independent constraint hypothesis. The second possibility is that an IC-based measure is better at capturing syntactic complexity compared to a DL-based measure. Thus, we also investigate the DLM as an independent constraint hypothesis, that is, whether dependency length is minimized independently of the constraint on intervener complexity. In sum, we test three related hypotheses: (a) ICM hypothesis, (b) ICM as an independent constraint hypothesis, and (c) DLM as an independent constraint hypothesis.

In order to test these hypotheses, we conduct a cross-linguistic corpus study where we compare the real trees attested in dependency treebanks with random baseline trees that match the real trees in certain properties. Such a methodology has previously been successfully employed to demonstrate the cross-linguistic validity of DLM (e.g., Futrell et al., 2015; Liu, 2008; Liu et al., 2017). For the purpose of this study, we introduce novel random baselines that are more restrictive compared to the baselines used previously. For instance, to evaluate whether intervener complexity is minimized independent of constraint on dependency lengths, we generate baseline trees controlled for the distribution of dependency lengths and compare them with the real trees in terms of intervener complexity.

The article is arranged as follows: In Section 2, we discuss the baselines and statistical methods used for testing the three hypotheses. In Section 3, we discuss the results for each hypothesis. We discuss the implications of the results in Section 4. Finally, we conclude the article in Section 5.

## MATERIALS AND METHODS

### Random Baselines

We employ six random baselines to test the hypotheses stated in the previous section. Each baseline controls for a particular set of tree properties relevant to the hypothesis.

Random baseline trees are generated by sampling from a uniform distribution over either random tree structures or random linear arrangements. We apply further constraints (like dependency length constraint) on these trees using rejection sampling to achieve the required sample for each baseline. We try to generate one baseline tree for each tree in the dependency treebank.

In all the baselines discussed below, we control the rate of crossing dependencies. In other words, baseline trees match the real trees in the number of crossing dependencies.^2^ Since crossing dependencies are rare in natural languages (Straka et al., 2015), random trees with large number of crossings tend to be dramatically different from real trees. Controlling for the rate of crossing, therefore, ensures a more strict baseline by preventing certain unrealistic structural configurations.

**Figure 2.**
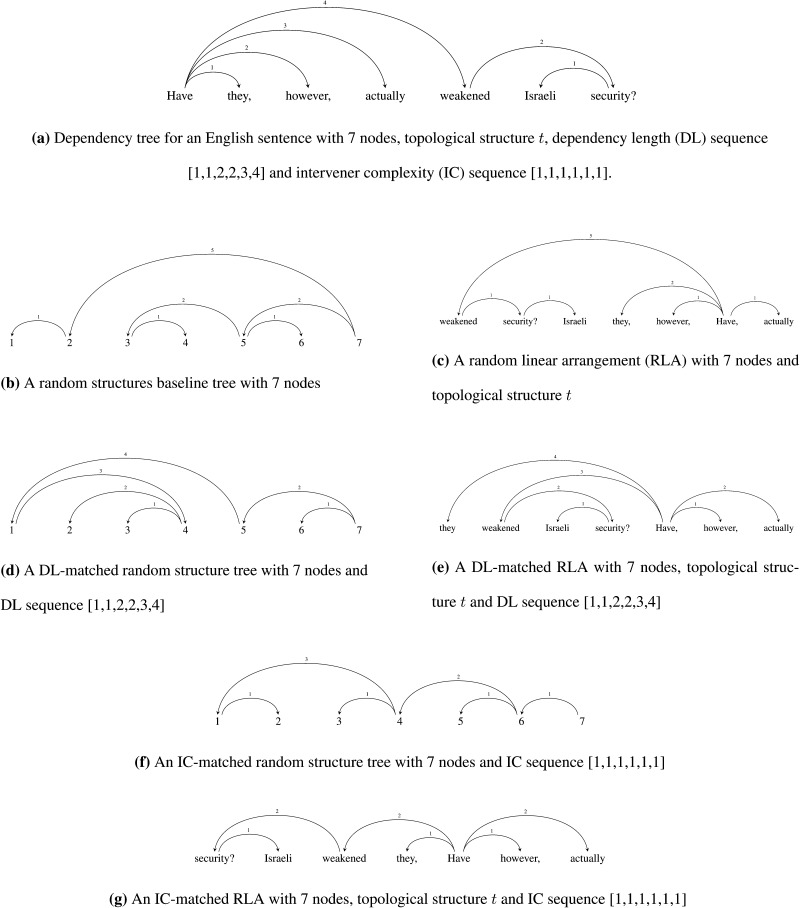
Sample trees for various random baselines corresponding to a dependency tree from an English treebank.

The ICM hypothesis is tested using the random structures baseline and the random linear arrangements (RLAs) baseline. In order to generate a *random structures baseline* tree for a given real language tree, we first compute the number of nodes, that is, sentence length, and the number of crossing dependencies in the real tree. Then using Prüfer codes (Prüfer, 1918), we sample trees from a uniform distribution over tree structures of a given number of nodes. Sampled trees that match with the number of crossings in the real trees are accepted as valid samples for the baseline. Hence, the random trees generated for this baseline are matched with real trees for the sentence length and the number of crossing dependencies. Figure 2(b) shows a random structure tree corresponding to a tree for a real sentence attested in a treebank—Figure 2(a). The RLAs baseline trees are sampled from a uniform distribution over all random linearizations of a given tree structure *t*. Compared to the random structures baseline, the RLA baseline preserves all the topological properties such as arity,^3^ tree depth, hubbiness, and so on, in addition to sentence length and number of crossings. This makes the RLA baseline more conservative than the random structures baseline (put differently, compared to the random structure trees, they are more similar to the real trees). RLAs are generated by permuting the order of the nodes in a real tree such that the dependency relations among the nodes are preserved. If a sampled tree matches the number of crossings in the real tree, it is accepted as a valid sample for the baseline. Figure 2(c) shows a sample RLA corresponding to a real tree in Figure 2(a).

In order to test the ICM as an independent constraint hypothesis, the random structures baseline trees and RLAs discussed above are further constrained by only selecting those baseline trees where the sequence of dependency lengths matches with the corresponding real tree. In other words, the baseline trees are obtained by restricting the dependency length distribution in the random structures and RLA trees. These baselines are termed respectively as DL-matched random structures baseline and DL-matched RLAs. Figures 2(d) and 2(e) show DL-matched random structure and DL-matched RLA, respectively, corresponding to a real tree in Figure 2(a). Note that, since these baselines control the dependency length sequence, they allow for a comparison of intervener complexity between the real trees and baseline trees *independent* of the influence of the dependency length distribution and topological properties like arity, and so on. In other words, any difference in intervener complexity between the real trees and DL-matched random structures baseline or DL-matched RLAs cannot be attributed to DL.

On similar lines, the DLM as an independent constraint hypothesis can be tested using the IC-matched random structures baseline and the IC-matched RLAs. These trees are sampled by restricting the intervener complexity distribution in the random structure and RLA trees, respectively. Figures 2(f) and 2(g) respectively show IC-matched random structure and IC-matched RLA corresponding to a real tree in Figure 2(a). We again note that, since these baselines control the IC sequence, they allow for a comparison of dependency length between the real trees and baseline trees *independent* of the influence of the intervener complexity and topological properties like arity, and so on. A summary of all the baselines can be found in Table 1.

**Table 1.** An overview of all six baselines.

**Random baseline**	**Controlled tree property**			
	**Sentence length**	**DL-sequence**	**IC-sequence**	**Tree topology**
Random structures baseline	✓	–	–	–
Random linear arrangements	✓	–	–	✓
DL-matched random structures	✓	✓	–	–
DL-matched RLAs	✓	✓	–	✓
IC-matched random structures	✓	–	✓	–
IC-matched RLAs	✓	–	✓	✓

*Note*. DL = dependency length; IC = intervener complexity; RLA = random linear arrangement. Tree topology controls for arity and depth.

The baselines mentioned above have the advantage of being quite constrained and therefore allow us to test various hypotheses rigorously. For example, the IC-matched RLA is a very conservative baseline as it controls both the topological properties such as arity, depth, number of crossings, as well as the intervener complexity distribution. This baseline will be used to test if there is any difference in dependency length distribution between real trees and baseline trees when the intervener complexity is the same in the real and random trees. While the above baseline allows us to test the DLM as an independent constraint hypothesis rigorously, its complexity makes the generation process of such baseline trees prohibitively slow. This is because we are controlling many properties of the baseline trees using rejection sampling. Therefore, we take sentences up to length 12 in this work. We discuss the issue of generalizability of our results in Section 4.

### Data

We use Surface-Syntactic Universal Dependencies (SUD) treebanks (version 2.4) (Gerdes et al., 2018, 2019) to perform all the analyses. We use the data of 54 languages. This set was obtained after excluding the treebanks for languages with fewer than 500 sentences and treebanks for ancient languages such as Latin, Ancient Greek, Sanskrit, Old Church Slavonic, Old Russian, and Old French. Our choice of SUD for the reported analysis is motivated by the widespread assumptions regarding syntactic representation in sentence processing research. In particular, this research subscribes to sentential representations consistent with modern linguistic theories (e.g., Bresnan, 1982; Chomsky, 1995; Hudson, 1984; Mel’čuk, 1988; Pollard & Sag, 1994) where function words are held to be syntactic heads (cf. Dillon, 2011; Gibson, 1998; Lewis & Vasishth, 2005). See Osborne and Gerdes (2019) for a detailed exposition on the syntactic assumptions in the SUD representation.

We compare the real trees attested in SUD treebanks with the baseline trees to test different hypotheses. As stated earlier, we take sentences up to length 12 in this work.

### Statistical Method

We want to test whether the distribution of intervener complexity or dependency length is significantly different between real trees and the baseline trees. In order to do this, we fit linear mixed-effect models (Bates et al., 2015) with varying intercepts and random slope adjustments for languages using the lme4 package in R (R Core Team, 2020).

Suppose *IC*_*ij*_ is the mean intervener complexity for *i*^*th*^ sentence of the *j*^*th*^ language, *S*_*ij*_ is the length of *i*^*th*^ sentence of the *j*^*th*^ language, *R*_*ij*_ is a dummy variable that encodes whether the sentence is a real tree (as 1) or a baseline tree (as 0), *β*_0_ is the intercept term, *β*_1_ and *β*_2_ are the slope terms for the main effect of sentence length and real/baseline variable respectively, *β*_3_ is the interaction term, *u*_0,*j*_ is the random intercept adjustment for *j*^*th*^ language, *u*_1,*j*_, *u*_2,*j*_ and *u*_3,*j*_ are random slope adjustments for the *j*^*th*^ language. The model to predict *IC*_*ij*_ is shown below

$${IC}_{ij}=\left(\beta_{0}+u_{0,j}\right)+\left(\beta_{1}+u_{1,j}\right)S_{ij}+\left(\beta_{2}+u_{2,j}\right)R_{ij}+\left(\beta_{3}+u_{3,j}\right)S_{ij}\cdot R_{ij}+\epsilon$$ (1)

Similarly, the model to predict mean dependency length for *i*^*th*^ sentence of the *j*^*th*^ language is shown below.

$${DL}_{ij}=\left(\beta_{0}+u_{0,j}\right)+\left(\beta_{1}+u_{1,j}\right)S_{ij}+\left(\beta_{2}+u_{2,j}\right)R_{ij}+\left(\beta_{3}+u_{3,j}\right)S_{ij}\cdot R_{ij}+\epsilon$$ (2)

For IC-related hypotheses, the dependent variable is the intervener complexity; for DL-related hypotheses, the dependent variable is dependency length. We check the interaction effect estimate $\hat{\beta }$_3_ to test whether the data supports our hypotheses regarding ICM and DLM.

The interaction effect estimate $\hat{\beta }$_3_ captures to what extent does the intervener complexity (or dependency length) grows slower in real trees compared to baseline trees with respect to sentence length. As an illustration, in order to test the ICM hypothesis, we check whether the growth of intervener complexity with respect to sentence length is significantly slower in real trees compared to random structure trees.

We note that the interaction parameter *β*_3_ is the effect of interest for testing our hypotheses because an aggregate difference in dependency length or intervener complexity between real trees and baseline trees (i.e., the main effect) could be subject to inaccuracies as the dependencies are mixed from different sentence lengths (see Ferrer-i Cancho & Liu, 2013; Futrell et al., 2015). In using the interaction effect for interpreting our results, we follow the recommendation in Ferrer-i Cancho and Liu (2013) that dependency length should be considered as a function of sentence length.

In addition to running the analysis on data for all the languages, we also tested the hypotheses individually for each language. While doing so, we remove the random intercept and slope adjustment for languages.

### Prediction

Recall that the ICM hypothesis is tested with intervener complexity as the dependent variable and uses the random structure and random linear arrangements baseline trees. The ICM as an independent constraint hypothesis is tested with intervener complexity as the dependent variable and uses the DL-matched random structure trees and DL-matched RLAs. Finally, the DLM as an independent constraint hypothesis is tested with dependency length as the dependent variable and uses the IC-matched random structure trees and IC-matched RLAs.

Each hypothesis predicts that the relevant dependent measure (IC or DL) grows slower in real language trees with respect to sentence length compared to the respective baseline. In particular, the ICM hypothesis predicts that the intervener complexity should grow slower in real language trees with respect to sentence length compared to random structure baseline trees and random linear arrangements. Similarly, the ICM as an independent constraint predicts that the intervener complexity should grow slower in real language trees with respect to sentence length compared to DL-matched random structure trees and DL-matched RLAs. Finally, the DLM as an independent constraint hypothesis predicts that the dependency length grows slower in real language trees with respect to sentence length compared to IC-matched random structure trees and IC-matched RLAs.

Therefore, if the estimated interaction effect coefficient $\hat{\beta }$_3_ is negative (see Equations 1, 2), it would be evidence in support for a particular hypothesis.

## RESULTS

**Figure 3.**
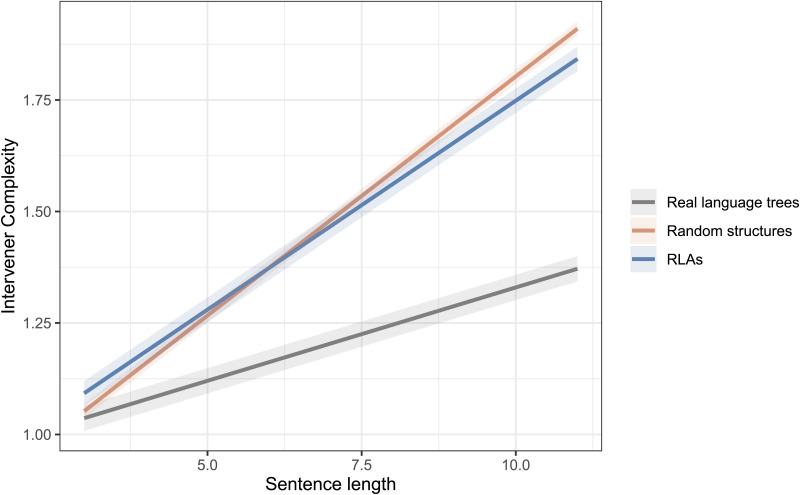
Fitted models showing the growth of intervener complexity with respect to sentence length in real language trees compared to random structure trees and random linear arrangements (RLAs).

With regard to the ICM hypothesis, Figure 3 shows the distribution of intervener complexity with respect to sentence length in real trees attested in treebanks and random baseline trees. Table 2 shows the estimates from the fitted linear-mixed models.^4^ We find that the average intervener complexity grows much slower in real language trees compared to random structures baseline trees ($\hat{\beta }$_3_ = −0.17, *t* value = −24.5) and random linear arrangements ($\hat{\beta }$_3_ = −0.13, *t* value = −19.7). The notes S7 and S8 in the Supplemental Materials show the language-specific analyses for the hypothesis.

**Table 2.** ICM hypothesis: Estimates from the fitted linear-mixed models for random structures baseline and random linear arrangements.

	**Random structures baseline**			**Random linear arrangements**		
	**Estimate**	***SE***	***t* value**	**Estimate**	***SE***	***t* value**
Intercept	1.48	0.008	172.96*	1.52	0.013	116.89*
S.length	0.29	0.004	71.07*	0.22	0.004	47.25*
Real	−0.28	0.015	−19.06*	−0.29	0.019	−15.40*
S.length:Real	−0.17	0.007	−24.46*	−0.13	0.006	−19.73*

*Note*. S.length = sentence length.

**Figure 4.**
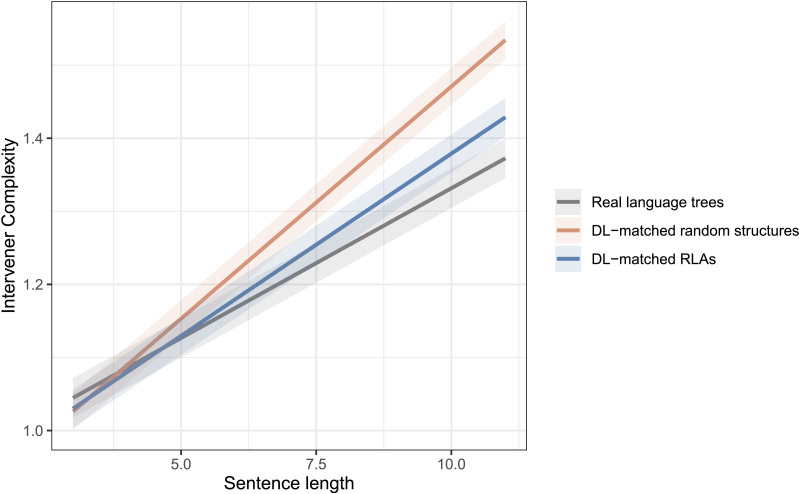
Fitted models showing the growth of intervener complexity with respect to sentence length in real language trees compared to dependency length (DL)-matched random structures and DL-matched random linear arrangement (RLAs).

A similar trend is observed with regard to the **ICM as an independent constraint hypothesis**, see Figure 4. Table 3 shows the estimates from the fitted linear-mixed models. The effect was found to be significant for both DL-matched random structures ($\hat{\beta }$_3_ = −0.03, *t* value = −6.4) and DL-matched RLAs ($\hat{\beta }$_3_ = −0.02, *t* value = −4.8).^5^

**Table 3.** ICM as an independent constraint: estimates from the fitted linear-mixed models for DL-matched random structures and DL-matched RLAs.

	**DL-matched random structures**			**DL-matched RLAs**		
	**Estimate**	***SE***	***t* value**	**Estimate**	***SE***	***t* value**
**Intercept**	1.19	0.009	132.85*	1.24	0.012	96.27*
**S.length**	0.15	0.007	19.54*	0.11	0.007	15.99*
**Real**	−0.03	0.003	−10.80*	−0.02	0.004	−4.91*
**S.length:Real**	−0.03	0.004	−6.40*	−0.02	0.004	−4.82*

*Note*. DL = dependency length; ICM = intervener complexity minimization hypothesis; RLA = random linear arrangement; S.length = sentence length.

**Figure 5.**
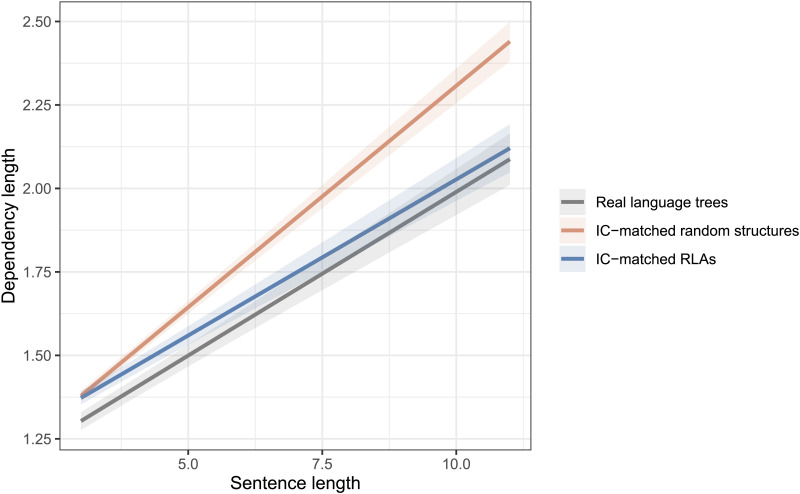
Fitted models showing the growth of dependency length with respect to sentence length in real language trees compared to intervener complexity (IC)-matched random structures and IC-matched random linear arrangement (RLAs).

Finally, with regard to DLM as an independent constraint hypothesis, the average dependency length grows significantly slower in real trees compared to IC-matched random structures ($\hat{\beta }$_3_ = −0.07, *t* value = −12.9). However, this pattern does not hold for IC-matched RLAs—the dependency length with respect to sentence length *does not* grow slower in real language trees compared to that in IC-matched RLAs ($\hat{\beta }$_3_ = 0.01, *t* value = 3.5). See Figure 5 and Table 4 for details.^6^

**Table 4.** DLM as an independent constraint: Estimates from the fitted linear-mixed models for IC-matched random structures and IC-matched RLAs.

	**IC-matched random structures**			**IC-matched RLAs**		
	**Estimate**	***SE***	***t* value**	**Estimate**	***SE***	***t* value**
**Intercept**	1.85	0.014	131.97*	1.81	0.022	79.50*
**S.length**	0.34	0.009	36.85*	0.22	0.010	22.31*
**Real**	−0.19	0.009	−21.71*	−0.04	0.005	−8.17*
**S.length:Real**	−0.07	0.005	−12.96*	**0.01**	**0.003**	3.51*

*Note*. DLM = dependency length minimization; IC = intervener complexity; RLA = random linear arrangement; S.length = sentence length.

## DISCUSSION

Our first key finding is that, cross-linguistically, the complexity of the linguistic material (measured as syntactic heads) intervening a syntactic dependency in treebank sentences is minimized. Our second key finding is that this minimization of intervener complexity holds even when the dependency length distribution is controlled in the random baseline trees. Finally, and most surprisingly, the results show that dependency length in real trees is not minimized against a baseline controlled for IC-distribution and topological structure of the tree. Together the results suggest that, cross-linguistically, intervener complexity captures syntactic complexity better than DL. Table 5 provides a summary of the results.

**Table 5.** Summary of evidence for each hypothesis.

**Random baseline**	**Evidence for hypothesis**		
	**ICM hypothesis**	**ICM as Independent Constraint**	**DLM as Independent Constraint**
Random structures baseline	✓	–	–
Random linear arrangements	✓	–	–
DL-matched random structures	–	✓	–
DL-matched RLAs	–	✓	–
IC-matched random structures	–	–	✓
IC-matched RLAs	–	–	✗

*Note*. ✓ means a baseline furnished evidence for tested hypothesis, ✗ means a baseline did not furnish any evidence for the hypothesis, – signifies not relevant; ICM = intervener complexity minimization hypothesis; DL = dependency length; IC = intervener complexity; RLA = random linear arrangement.

### Is DLM Epiphenomenal?

Results show that an optimal linear arrangement for minimizing intervener complexity could, in turn, minimize DL. How can we interpret this finding?

**Figure 6.**
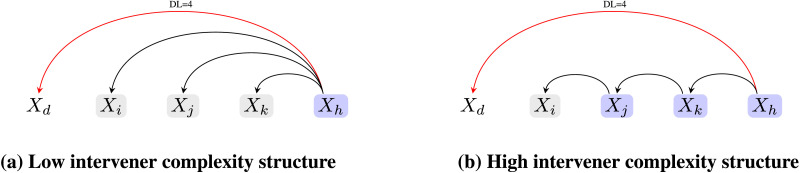
**A schematic showing that a given dependency length (e.g., length = 4 for the dependency *X*_*h*_ → *X*_*d*_) can be obtained by two types of structures.** Low intervener complexity structure (a) has higher arity and few heads. High intervener complexity structure (b) has low arity and more number of intervening heads.

We begin by noting that a particular dependency length can result from two types of intervening structures: *(a) Low intervener complexity structure* having more intervening dependents and fewer intervening heads, or *(b) High intervener complexity structure* having more intervening heads and fewer intervening dependents.^7^ Figure 6 shows the two structures; the observed dependency length of *X*_*h*_ → *X*_*d*_ in structure (a) is driven entirely by intervening dependents, while in (b), it is primarily driven by intervening heads. Notice that a low intervener complexity structure requires a high arity for at least one of the nodes in the structure (e.g., *X*_*h*_ in Figure 6a).

Given these two intervener complexity configurations, results for the *ICM as an independent constraint* hypothesis show that cross-linguistically a low intervener complexity structure is preferred over a high intervener complexity structure. Recall that the hypothesis was tested using DL-matched baselines where the distribution of dependency length is identical to the real trees. The results for this hypothesis, therefore, are not driven by dependency length–related constraints. We now assess the results for DLM as an independent constraint hypothesis in the light of the constraint that natural languages prefer low intervener complexity structures.

#### DL Minimization in Real Trees Against IC-Matched Random Structures

**Figure 7.**
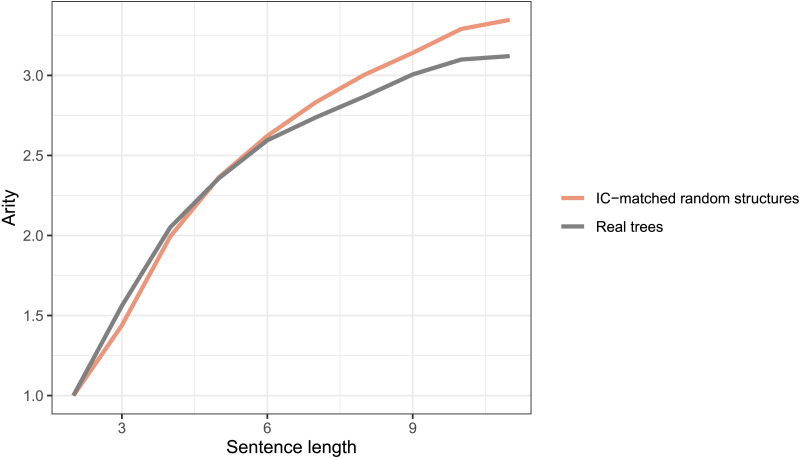
**The distribution of tree arity in real trees and intervener complexity (IC)-matched random structures.** IC-matched random structures use flexibility in topological structure to posit higher arity and hence longer dependency distance than real trees.

Assuming the ICM constraint on real language trees, IC-matched random structures trees cannot posit syntactic configurations with high-intervener complexity (see Figure 6). However, there is no restriction on the topological structure of these random trees.^8^ Consequently, these random trees can have more instances of structures with high arity compared to real trees. As a result, they could still posit longer dependencies in spite of low-intervener complexity configurations (see Figure 6). Figure 7 shows that arity in IC-matched random structures is higher than real trees, especially for longer sentences. This demonstrates that arity distribution in real trees is an important determinant of dependency length.

#### No Evidence for Dependency Length Minimization in Real Trees Against IC-matched RLAs

Compared to the IC-matched random structures trees, the possibility to posit longer dependencies due to flexibility in topological structure gets severely restricted in IC-matched RLAs.^9^ As a consequence, the two mechanisms that can drive long dependencies (see Figure 6) are less accessible here. Consequently, IC-matched RLAs do not show conclusive evidence for dependency length minimization in real trees. This suggests that, together, the constraints on intervener complexity and constraints on topological structures of trees, like arity, could determine the distribution of dependency length in natural language.

#### Asymmetry in Constraints on Intervener Complexity Versus Dependency Length

**Figure 8.**
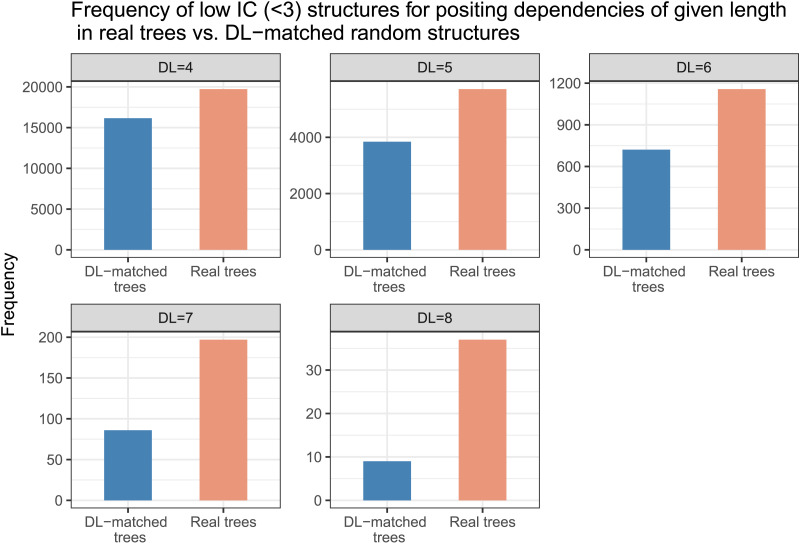
**The number of low intervener complexity (IC) (≤ 2) structures in real trees and dependency length (DL)-matched random structures at each dependency length.** Compared to the baseline trees, the real trees tend to use low IC structures for positing longer dependencies.

**Figure 9.**
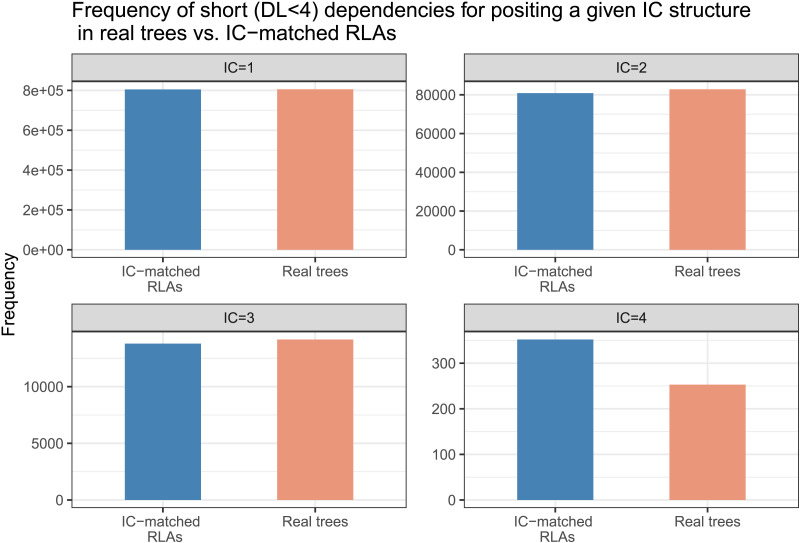
**The frequency of short (dependency lenth—DL ≤ 3) dependencies in real trees versus intervener complexity (IC)-matched random linear arrangement (RLAs) at each intervener complexity.** Compared to the baseline trees, the real trees do not show preference for short dependencies for positing a given IC structure. Figure shows up to IC 4, because high IC (> 4) structures cannot be achieved by short (DL ≤ 3) dependencies.

**Figure 10.**
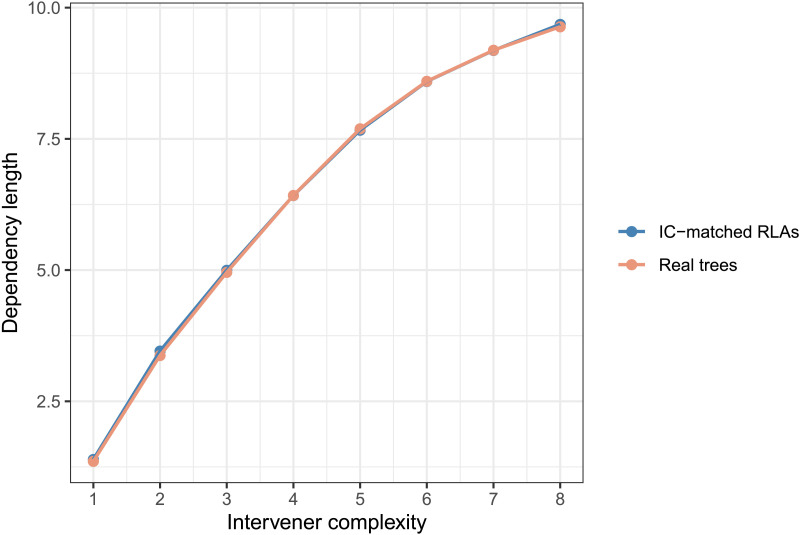
The average dependency length at each intervener complexity (IC) in real trees versus IC-matched random linear arrangement (RLAs).

In order to understand the nature of structures preferred by real trees for positing dependencies of a given IC or a given DL, we did an exploratory analysis. We note the following:

1. For positing dependencies of a given length, the real trees use low-IC structures more frequently compared to the DL-matched baseline trees (see Figure 8). This implies that real trees prefer low-IC structures regardless of dependency length. This low-IC tendency in real trees becomes even more stronger for longer dependencies.
2. By contrast, real trees do not show much preference for low-DL structures when compared with IC-matched RLAs (see Figure 9). For positing structures with a given IC, the real trees choose almost as many short dependencies as the baseline trees. Moreover, the real trees and IC-matched RLAs have the same average DL for a given intervener complexity (see Figure 10).

The above points suggest an asymmetry in constraints on IC versus DL in real trees: Compared to a baseline controlled for DL, the real trees prefer low IC structures; but compared to the RLAs controlled for IC, the real trees do not show much preference for shorter dependencies. This asymmetry supports the ICM as an independent constraint hypothesis, but does not support the DLM as an independent constraint hypothesis.

### Notes on Methodology and Limitations of the Current Work

As stated earlier, multiple corpus-based work (e.g., Futrell et al., 2015; Gildea & Temperley, 2010; Liu, 2008) have previously provided evidence for DLM cross-linguistically using the method similar to the one employed in the current study. Given that the methodology involves the comparison of real trees with random baseline trees, the nature of these baseline trees becomes critical. Most previous work (e.g., Futrell et al., 2015; Liu, 2008) use baselines akin to the random structures baseline and RLAs. In the current work, we wanted to directly assess the evidence for the independence of two constraints—whether a certain constraint *X* on real trees holds independent of another constraint *Y*. This required us to compare real trees against baseline trees that were generated under constraint *Y*. Therefore, compared to previously used random structures or RLAs, the baselines employed in the current work are strongly constrained. For example, to test whether ICM occurs independent of DLM, we compare real trees against baseline trees that have constraints on dependency-length distribution and tree topology. In addition, unlike baselines in previous work, which either had only noncrossing trees or an unreasonably large number of crossing dependencies, the baselines in the current work controlled for the number of crossings. However, controlling for multiple properties makes the generation process of these baselines very slow. Due to this reason, we have provided evidence for the role of intervener complexity and arity in determining syntactic complexity in natural languages using various baselines for sentence length < 12. So, while our baselines allow for a rigorous evaluation of various hypotheses, they are based on relatively short sentences. This could raise concerns regarding the generalizability of the current results. In order to assuage such concerns, below, we provide some observation of IC/arity patterns in real trees that suggests the results should hold for longer sentences as well.

**Figure 11.**
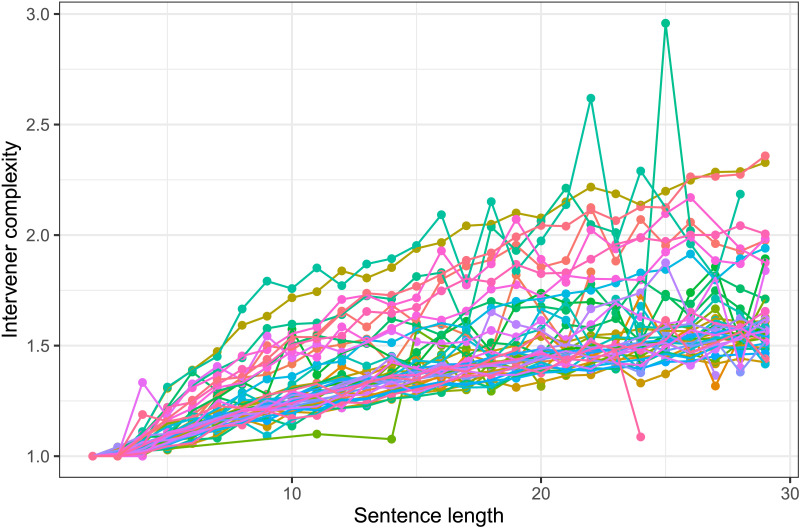
**Intervener complexity at various sentence lengths for various languages.** The figure shows that intervener complexity grows with sentence length at almost the same rate for short and long sentences cross-linguistically, which indicates that constraint on intervener complexity persists for longer sentences.

**Figure 12.**
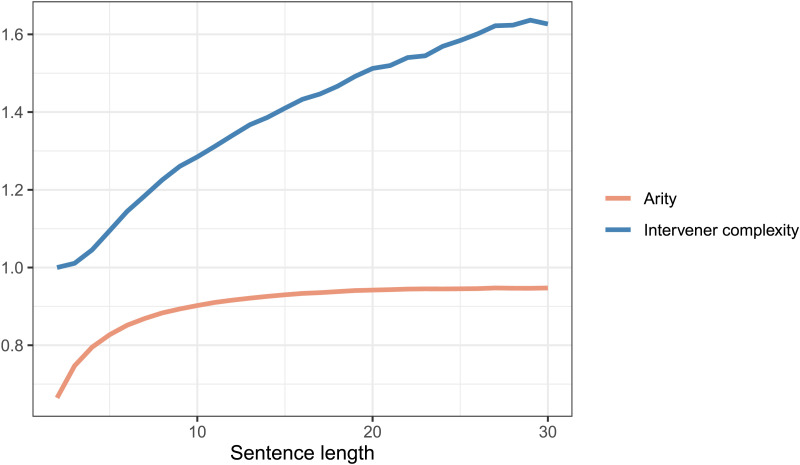
**The figure compares the rate of growth of intervener complexity and arity with respect to sentence length.** Intervener complexity grows at almost the same rate for short and long sentences, while tree arity becomes increasingly restricted for longer sentences.

**Figure 13.**
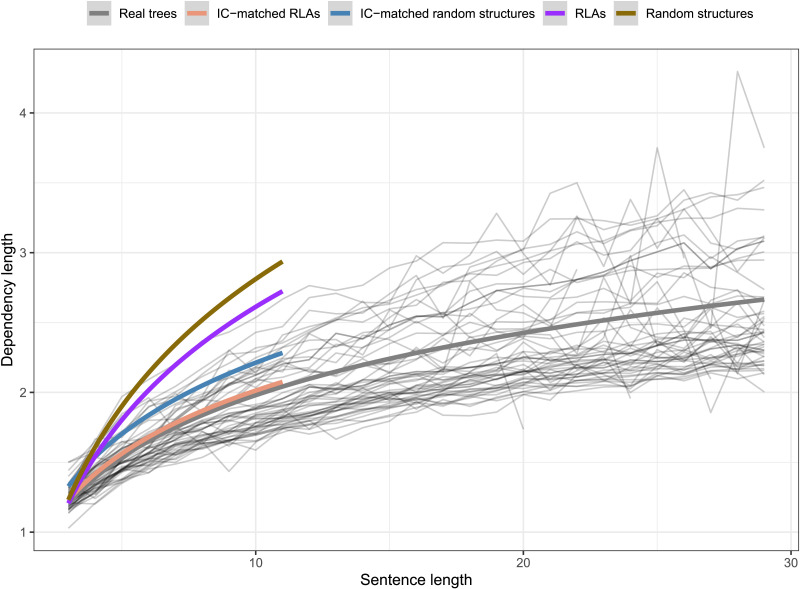
**Growth of dependency length with respect to sentence length in real trees versus baseline trees.** Light gray lines represent various real languages, thick gray line represents average growth across real trees, thick colored lines represent random baseline trees of sentence length less than 12.

1. Figure 11 shows that the rate of intervener complexity growth with sentence length is almost the same for short and long sentences. This suggests that the constraint on intervener complexity persists for longer sentences.
2. Figure 12 shows that arity in real sentences becomes severely restricted in longer sentences, while intervener complexity grows at almost the same rate for short and long sentences. This implies that IC-matched RLAs—the baseline trees that match in arity and intervener complexity with real trees—would have much stronger restrictions on dependency length in longer sentences. This is because, as discussed earlier, positing a longer dependency requires either a high arity or a high IC, but high-arity configurations get severely restricted in longer sentences. This would predict that dependency length in real trees would grow at almost the same rate or faster than IC-matched RLAs. Figure 13 shows the rate of growth of dependency length in real trees up to sentence length 30 and in baseline trees up to sentence length 11.

The above observations (and related figures) show that compared to short sentences, the ICM/arity effects in real trees are even stronger in longer sentences. This provides a reasonable basis to believe that the current results will hold for long sentences. We plan to take up baseline generation for long sentences in the near future.

Additional concerns regarding our conclusions could be that (a) shorter sentences might belong to nonrepresentative text in the corpus such as headlines, article headings, and (b) we do not have enough power to accept the null hypothesis regarding DLM as an independent constraint. For (a), we did an additional analysis by extracting clauses of length up to 12 words from longer (> 12 words) sentences and compared them with corresponding IC-matched baseline trees.^10^ We were able to replicate the results for DLM as an independent constraint hypothesis: dependency length grows significantly slower in real trees from clausal data compared to IC-matched random structures but not when compared with IC-matched RLAs (see Note S5 in the Supplemental Materials). For (b), we did a Bayes factor analysis. We find moderate to strong evidence in the favor of the null hypothesis (see Note S4 in the Supplemental Materials for detailed results). The result suggests that the confidence in accepting the null hypothesis regarding DLM as an independent constraint should be reasonably high.

Finally, the lack of evidence for the DLM as an independent constraint in this work has been based on a lack of significant interaction in the right direction (see Table 4). However, we do find a main effect of tree type (real vs. random): the average dependency length is shorter in real trees compared to IC-matched RLAs at each sentence length (all *t* values < −2). As pointed out by an anonymous reviewer, this pattern goes against our claim that DLM could be a consequence of constraint on IC and tree topology. Our choice of using the interaction effect to test the hypothesis is based on one of the definitive, large-scale corpus investigation of dependency length minimization (Futrell et al., 2015) that uses the interaction effect estimate to argue for the DLM hypothesis. Given the importance of the claims in Futrell et al. (2015), it is imperative that a comparative study of DLM against a competing hypothesis should also use a similar methodology. However, in the context of our last claim about potential nonindependence of DLM, different conclusions can be drawn based on the estimates of main effect (at each sentence length) and the interaction effect. Considering this methodological issue, we cannot conclusively argue that DLM might arise due to constraint on IC and arity restrictions. The only certain conclusion from our study is that ICM is an independent constraint on language while DLM may or may not be epiphenomena of ICM. Our additional analyses show that ICM is indeed a stronger constraint compared to DLM in determining the distribution of word order and syntactic choices in natural languages.

The current work, therefore, shows that, in shorter sentences, ICM is an independent constraint on natural languages. On the other hand, we do not find any conclusive evidence for DLM as an independent constraint suggesting that DLM might arise as a consequence of ICM and arity restrictions. However, it remains a possibility that our conclusions are driven by methodological idiosyncrasies (i.e., we interpreted the interaction effects only) and/or nature of the data (i.e., we used only shorter sentences). At the very least, the current work conclusively shows ICM and arity restrictions to be an equally important determinant of syntactic complexity as DLM.

### Measuring Syntactic Complexity

Building syntactic structures efficiently is a key aspect of language processing. Numerous research has highlighted that simple and easier structures are preferred during both comprehension (e.g., Ferreira et al., 2002; Ferreira & Patson, 2007; Fodor & Inoue, 2000; Frazier, 1985; Gibson, 1998; Lewis & Vasishth, 2005) and production (e.g., Bock & Warren, 1985; Ferreira, 1991; Gibson et al., 2019; Hahn et al., 2020; Kurumada & Jaeger, 2015; MacDonald, 2013). Since syntactic heads can be assumed to be central regions of structural integrations during processing, it is not surprising that these processing-intensive units should be avoided while building a dependency.

Quantifying complexity as intervening heads is consistent with previous proposals where the number of nonterminal nodes of a phrase structure tree has been assumed to be an important determinant of processing difficulty^11^ (e.g., Ferreira, 1991; Frazier, 1985; Miller & Chomsky, 1963; Yngve, 1960). The current work also highlights the key role of arity in determining syntactic complexity. Results show that real trees have lower arity than that found in baselines such as IC-matched RLA. This is not surprising when we consider that the syntactic requirements of heads are constrained in natural languages. For example, in English, it will be rare to find verb lemmas where the number of arguments would be more than three. The current work suggests that linguistic constraints related to a head’s requirements (e.g., verb’s argument structure) are important determinants of dependency length.

Overall, considerable previous work has designated phrasal complexity and number of words to be two independent ways to quantify syntactic complexity in natural languages (Ferreira, 1991; Szmrecsányi, 2004; Wasow, 1997; Wasow & Arnold, 2003). However, no previous work, to our knowledge, has tested if one of these measures is better at capturing complexity when the other is held constant. The current work introduces a method to evaluate the relative performance of a complexity measure cross-linguistically using corpus data (also see Yadav et al., 2019). Using our method, one can test whether a constraint on measure *X* occurs independently of a constraint on measure *Y*. We can do this by comparing the distribution of *X* in real trees with baseline trees matched in *Y* with real trees. Using this method, we tested the independence of constraints on intervening heads and constraints on intervening words. We found that the number of intervening heads is a better measure of complexity than the number of intervening words. Thus, our methodology provides a principled way to evaluate new complexity measures against existing ones.

With regard to various heads intervening a dependency, the ICM hypothesis predicts a greater avoidance of high-processing heads (i.e., those that involve a larger number of syntactic integrations) compared to low-processing heads. Given varying syntactic constraints, it is reasonable to assume a differential processing cost at various heads. For example, verbal heads would typically involve more integrations than adjectival heads (cf. Frazier, 1985; Gibson, 1998; Gibson & Thomas, 1999; Miller & Chomsky, 1963; Yngve, 1960). Future work will extend the current work by reformulating the intervener complexity measure to capture both the number and the type of intervening heads.

### Syntactic Complexity and Linguistic Typology

The current work suggests that the number of intervening heads could be a better measure to quantify syntactic complexity compared to the number of intervening words. Could typologically distinct languages differ in their distribution of intervening heads and words? More importantly, could the results for ICM/DLM as an independent hypothesis differ based on language typology?

**Figure 14.**
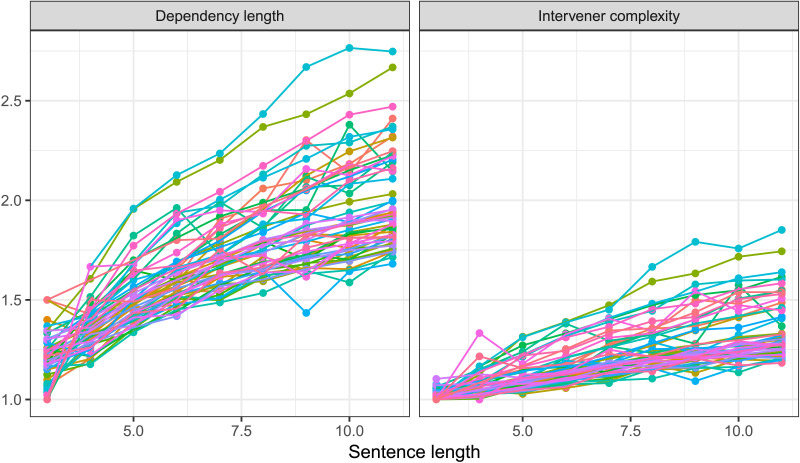
**The distribution of dependency length and intervener complexity with respect to sentence length across language.** Intervener complexity shows less variability across languages and across sentence length compared to dependency length.

We did an additional analysis to test these questions, specifically testing if (a) distribution of intervening words/heads differ in Subject-Object-Verb (SOV) versus Subject-Verb-Object (SVO) languages, and (b) if the results for ICM/DLM as an independent hypothesis on aggregated data differ for SOV versus SVO languages. Regarding (a), results show that the number of intervening heads, as well as the number of intervening words, are more in SOV languages compared to SVO languages. Interestingly, a recent cross-linguistic corpus study by Yadav et al. (2020) shows that the number of intervening heads is highly constrained across languages, and this constraint shows less variability compared to the number of intervening words (see Figure 14). Regarding (b), we find that both SOV and SVO languages show expected dependency length and intervener complexity minimization that was found in the aggregated data, that is, IC/DL grows significantly slower in real trees compared to random baseline trees (except IC-matched RLAs). At the same time, the effect of minimization is weaker in SOV language compared to SVO languages suggesting a degree of linguistic adaptability in SOV languages (cf. Levy & Keller, 2013; Vasishth et al., 2010; Yadav et al., 2020).^12^ Together these additional analyses suggest that results obtained on the aggregated data can be generalized to these typologically distinct languages.

## CONCLUSION

This work presents a corpus investigation to show that dependency length minimization as a cross-linguistic constraint is better operationalized as the minimization of the number of syntactic heads that intervene a dependency rather than as the minimization of the number of words. We use a novel method to demonstrate this result. In particular, we show that when real trees are compared with random trees that control for intervening heads (and other tree properties such as arity), there is no conclusive evidence for dependency length minimization (in terms of the number of words) in the real trees. On the other hand, when real trees are compared with random trees that control for dependency length and various tree properties, we find evidence for intervener complexity minimization. These results suggest that, compared to the number of words, intervener complexity could be a better measure to quantify cross-linguistic syntactic complexity.

## ACKNOWLEDGMENTS

We would like to thank the two anonymous reviewers for their comments. We also thank Richard Futrell for his comments on an earlier draft of the paper.

## AUTHOR CONTRIBUTIONS

HY: Conceptualization: Equal; Formal analysis: Lead; Methodology: Equal; Supervision: Equal; Visualization: Lead; Writing - Original Draft: Supporting; Writing - Review & Editing: Equal. SM: Formal analysis: Supporting; Visualization: Supporting; Writing - Review & Editing: Supporting. SH: Conceptualization: Equal; Methodology: Equal; Supervision: Equal; Writing - Original Draft: Lead; Writing - Review & Editing: Equal.

## Notes

^1^ Indeed, such a prediction will also hold for proposals that do not make a distinction between temporary storage and integrations (e.g., Lewis & Vasishth, 2005). Under such an account, increased structure building due to intervening heads will lead to retrieval difficulty of the dependent *X*_*d*_ due to time-driven decay (or similarity-based interference).^2^ A crossing dependency is formed when two dependencies cross each other. Formally, a dependency, *h* → *d* with *h* as the head and *d* as its dependent, is a crossing dependency if and only if there is at least one node, say *i*, that intervenes *h* and *d* such that *h* does not (directly or indirectly) dominate *i*.^3^ Arity of a node in a tree is defined as the number of dependents of that node.^4^ All the data and reproducible analysis files are available at https://osf.io/j975y/.^5^ The notes S9 and S10 in the Supplemental Materials show the language-specific analysis for the ICM as an independent constraint hypothesis.^6^ The notes S11 and S12 in the Supplemental Materials show the language-specific analysis for the DLM as an independent constraint hypothesis.^7^ Intervening dependents here mean the terminal dependents that intervene a dependency.^8^ Recall that IC-matched random structures trees match in intervener complexity distribution, however they do not control for topological properties (e.g., arity) of the real trees.^9^ Recall that IC-matched RLAs control for intervener complexity as well as topological properties (such as arity).^10^ We thank an anonymous reviewer for suggesting this method.^11^ Intervener complexity might also seem related to the *storage cost* metric proposed in Gibson (1998), but they are distinct. See Note S2 in the Supplemental Materials for more details.^12^ Note S6 in the Supplemental Materials provides detailed results for these analyses.

## Supplemental Materials

https://osf.io/j975y/
