# Supplementary material for: A Reappraisal of Dependency Length Minimization as a Linguistic Universal
Source: Open Mind (Camb). 2022 Sep 15;6:147–68. doi: 10.1162/opmi_a_00060 (PMC9692064; doi:10.1162/opmi_a_00060)
Supplement: Supplementary file 1 [file opmi-06-147-s001.pdf]

# Supplementary Materials

## A reappraisal of dependency length minimization as a linguistic universal

**Contents**


**Note S1**
**Random baselines** **2**

**Note S2**
**A note on intervener complexity** **5**

**Note S3**
**Linear-mixed models with language family as a random effect** **6**

**Note S4**
**Bayes factor-based evidence against DLM as an independent constraint hypothesis** **7**

**Note S5**
**Analysis using data from clauses taken from sentences longer than 12 words** **9**

**Note S6**
**Effect of word-order typology on IC/DL minimization: Linear-mixed models with linguistic typology as a predictor** **10**

**Note S7**
**Distribution of intervener complexity in real trees against random structure trees** **12**

**Note S8**
**Distribution of intervener complexity in real trees against random linear arrangements (RLAs)** **15**

**Note S9**
**Distribution of intervener complexity in real trees against DL-matched random structures** **18**

**Note S10**
**Distribution of intervener complexity in real trees against DL-matched RLAs** **21**

**Note S11**
**Distribution of dependency length in real trees against IC-matched random structures** **24**

**Note S12**
**Distribution of dependency length in real trees against IC-matched RLAs** **27**

**Note S13**
**Analysis using Universal Dependencies treebanks data** **30**

**Note S14**
**Languages used in the analysis** **31**

**References** **32**

## Note S1
## Random baselines

We employ six random baselines to test the three hypotheses, namely Intervener Complexity Minimization (ICM), ICM as an Independent Constraint and Dependency Length Minimization (DLM) as an Independent constraint hypothesis. Each baseline controls for a particular set of tree properties relevant to the hypothesis. For example, in order to test the **ICM as an Independent Constraint** hypothesis, we crucially control the distribution of dependency length in the baseline trees and compare the distribution of intervener complexity between the baseline trees and the real trees.

Random baseline trees are generated by sampling from a uniform distribution over either random tree structures or random linear arrangements. We apply further constraints (like dependency length constraint) on these trees using rejection sampling to achieve the required sample for each baseline. For example, DL-matched random structures are generated by first sampling from a uniform distribution over tree structures and then rejecting the trees that do not match in dependency length sequence with the corresponding real tree. We intend to generate one baseline tree for each tree in the dependency treebank.

We decided to control the rate of crossing dependencies for each baseline such that baseline trees should match the real trees in terms of the number of crossing dependencies. A crossing dependency is formed when two dependencies cross each other. Formally, a dependency, $h \to d$ with $h$ as the head and $d$ as its dependent, is a crossing dependency if and only if there is at least one node, say $i$, that intervenes $h$ and $d$ such that $h$ does not (directly or indirectly) dominate $i$. The rationale behind controlling the rate of crossings dependencies is to prevent an unrealistically large number of crossings in the random trees. The trees

with a large number of crossings are dramatically different in terms of dependency structure from real trees because crossing dependencies are rare in natural languages (Straka, Hajic, Straková, & Hajic jr, 2015). We do not want to make any additional assumption about why crossing dependencies are rare in natural languages; we make a safer assumption that the rarity of crossing dependencies is a constraint on natural language trees. Hence, each baseline controls for the number of crossing dependencies in addition to all other measures relevant for the hypothesis.

In the following sections, we describe the procedure of generating trees for each baseline corresponding to real trees attested in the dependency treebanks.

### Random structures baseline

Random structure baseline trees are sampled from a uniform distribution over tree structures with $n$ nodes, with a further restriction that the sampled tree must match the number of crossing dependencies in the corresponding real language tree. In order to generate a random structures baseline tree for a given real language tree, we first compute the number of nodes, i.e., the sentence length and the number of crossing dependencies in the real tree. Then using Prüfer codes (Prüfer, 1918), we sample trees from a uniform distribution over tree structures of a given number of nodes. Sampled trees that match the number of crossings in dependencies in the real trees are accepted as valid samples for the baseline. Hence, the random trees generated for this baseline are matched with real trees for the sentence length and the number of crossing dependencies.

### Random linear arrangements (RLAs) baseline

The random linear arrangements (RLAs) are sampled from a uniform distribution over all random linearizations of a given tree structure $t$, with a further constraint on the number of crossing dependencies. In order to generate a random linear arrangement for a real language tree, we first sample a tree by permutating the order of the nodes of the real tree such that the dependency relations among the nodes are kept the same. If a sampled tree matches the number of crossings dependencies in the real tree, it is accepted as a valid sample for the baseline.

Since dependency relations among nodes are preserved, RLAs control for all the topological properties such as arity, tree depth, hubbiness, etc., in addition to sentence length and number of crossings. This makes the RLA baseline slightly more conservative than the random structures baseline; a random structure tree could be biased for certain properties and may not make a good baseline for comparison with the real trees. This problem gets resolved in the RLAs.

### DL-matched random structures baseline

DL-matched random structures baseline is obtained by restricting the dependency length distribution in the random structures baseline trees. In order to generate a DL-matched random structure tree, we first sample a random structure tree and then accept the tree as a valid sample only if it matches the sequence of dependency lengths in the corresponding real tree. This baseline allows us to observe the distribution of intervener complexity independent of the influence, if any, of the dependency length distribution, as the baseline

trees have the same dependency length sequence as the real trees. Hence, any observed difference between real and random trees in terms of intervener complexity, here, cannot be a consequence of dependency lengths.

### DL-matched RLAs

DL-matched RLAs are obtained by restricting the dependency length distribution in the random linear arrangements of real trees. In order to generate a DL-matched RLA, we first sample a random linear arrangement of a real tree and then accept the tree as a valid sample only if it matches the sequence of dependency lengths in the corresponding real tree. This baseline allows the comparison of intervener complexity between real tree and baseline trees independent of the influence of the dependency length distribution.

### IC-matched random structure baseline

IC-matched random structure trees are sampled by restricting the intervener complexity distribution in the random structure trees. To generate an IC-matched random structure, we first generate a random structure tree (using the method described in section 3.1) and then apply rejection sampling to sample a tree only if it matches the sequence of intervener complexity in the corresponding real tree. We use this baseline to observe the dependency length distribution independent of the influence of the intervener complexity.

### IC-matched RLAs

An IC-matched RLA for a real tree with topological structure $t$ is generated by first sampling an RLA with topological structure $t$ (as described in section 3.2) and then accepting the RLA only if it matches the sequence of intervener complexity in the corresponding real tree. This is a very conservative baseline as it controls both the topological properties such as arity, depth etc. and the intervener complexity distribution. We use this baseline to test if there is any difference in dependency length distribution between real trees and baseline trees when the intervener complexity is the same in both.

## Note S2
## A note on intervener complexity

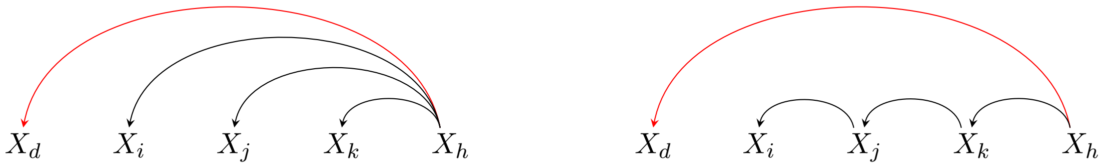


**(a)** IC = 1, DL = 4 **(b)** IC = 3, DL = 4

**Figure S1**

*Dependency structures with varying intervener complexity for $X_h \rightarrow X_d$. While the number of words (DL) that intervene $X_h \rightarrow X_d$ in tree (a) and tree (b) is the same, the two structures differ in the number of intervening heads (IC).*

Intervener complexity can be conceptualized as related to the storage cost component of the Dependency locality theory (DLT) (Gibson, 1998). At any stage during comprehension of a sentence, the storage cost metric at a word captures the syntactic heads required to complete the sentence grammatically. For example, in Figure S1(a), the storage cost at $X_j$ would be 1 as only one syntactic head ($X_h$) is required to complete the sentence grammatically. On the other hand, in Figure S1(b), the storage cost at $X_j$ would be 2 as two syntactic heads ($X_k$, $X_h$) are required to complete the sentence grammatically. DLT assumes that both storage cost and integration cost (for which dependency length can be thought of as an approximation) access the same pool of working-memory resources. This means that if the storage cost is high, there will be difficulty at the integration site.[1] This suggests that languages should minimize both the number and complexity of words that intervene syntactically related words. Intervener cost (IC) and storage cost (SC) are similar to the extent that syntactic heads are important to both the metric; the similarity ends there. IC is defined for a dependency, while SC is defined for a word. Consequently, with regard to Figure S1 (a),(b), the IC for $X_d \rightarrow X_h$ captures the processing cost while $X_d$ (and/or the prediction of $X_h$) has to be maintained in memory. The SC at $X_h$ in Figure S1 (a),(b) will be 0 for both the dependency structures and therefore, it does not capture the possibility of a processing difficulty at $X_h$ due to the processing of the complex intervening material in Figure S1(b). SC can, of course, capture the processing difficulty at $X_j$ and $X_k$ where integrations of $X_i$ and $X_j$ respectively need to take place while some heads are being actively maintained. In sum, IC is a measure to capture the processing difficulty of a dependency, and it is measured as the sum of the number of heads that intervene a dependency arc.

[1]From (Gibson, 1998, pp16), *Thus the greater the memory cost, the smaller the resources available to perform linguistic integrations, and the longer the integration steps will take.*

## Note S3
## Linear-mixed models with language family as a random effect

Let, $IC_{ij}$ be the (average) intervener complexity for sentence $i$ in language $j$. $R_{ij}$ be the dummy variable encoding whether the sentence is from real language or random baseline, $S_{ij}$ be the $i^{th}$ sentence of language $j$. $u_{0j}$, $u_{1j}$, $u_{2j}$, and $u_{3j}$ are random intercept and slope adjustments for language $j$. $w_{0k}$ is random intercept adjustment for language family $k$. $\beta_0$, $\beta_1$, $\beta_2$ and $\beta_3$ are the parameters associated with intercept, effect of sentence length, effect of real/random variable and interaction effect respectively.

$$IC_{ij} = (\beta_0 + u_{0j} + w_{0k}) + (\beta_1 + u_{1j})S_{ij} + (\beta_2 + u_{2j})R_{ij} + (\beta_3 + u_{3j})S_{ij} * R_{ij} \quad (1)$$

We check the estimate of interaction coefficient to $\beta_3$ to test the Intervener Complexity Minimization (ICM) hypothesis. If the ICM hypothesis is true, the $\hat{\beta_3}$ will be negative. $\hat{\beta_3}$ represents the extent to which intervener complexity grows slower in real trees compared to the baseline trees. We similarly test the Dependency Length Minimization (DLM) hypothesis for IC-matched baselines; average dependency length is used as the dependent variable to test the DLM hypothesis.

**Table S1**

*Estimates from the fitted linear-mixed models with language family as random effects.*

| **ICM hypothesis** | | | | | | |
|---|---|---|---|---|---|---|
| | **Random structures baseline** | | | **Random linear arrangements** | | |
| | **Estimate** | **Std. Error** | **t-value** | **Estimate** | **Std. Error** | **t-value** |
| **Intercept** | 1.48 | 0.008 | 172.97 * | 1.52 | 0.013 | 116.89 * |
| **S.length** | 0.29 | 0.004 | 71.07 * | 0.22 | 0.004 | 47.25 * |
| **Real** | -0.28 | 0.015 | -19.07 * | -0.29 | 0.019 | -15.40 * |
| **S.length:Real** | -0.17 | 0.007 | -24.47 * | -0.13 | 0.006 | -19.73 * |

| **ICM as an independent constraint** | | | | | | |
|---|---|---|---|---|---|---|
| | **DL-matched random structures** | | | **DL-matched RLAs** | | |
| | **Estimate** | **Std. Error** | **t-value** | **Estimate** | **Std. Error** | **t-value** |
| **Intercept** | 1.19 | 0.009 | 132.85 * | 1.24 | 0.012 | 96.27 * |
| **S.length** | 0.15 | 0.007 | 19.54 * | 0.11 | 0.007 | 15.99 * |
| **Real** | -0.03 | 0.003 | -10.80 * | -0.02 | 0.004 | -4.91 * |
| **S.length:Real** | -0.03 | 0.004 | -6.40 * | -0.02 | 0.004 | -4.82 * |

| **DLM as an independent constraint** | | | | | | |
|---|---|---|---|---|---|---|
| | **IC-matched random structures** | | | **IC-matched RLAs** | | |
| | **Estimate** | **Std. Error** | **t-value** | **Estimate** | **Std. Error** | **t-value** |
| **Intercept** | 1.85 | 0.014 | 131.97 * | 1.81 | 0.022 | 79.50 * |
| **S.length** | 0.34 | 0.009 | 36.85 * | 0.22 | 0.010 | 22.31 * |
| **Real** | -0.19 | 0.009 | -21.71 * | -0.04 | 0.005 | -8.17 * |
| **S.length:Real** | -0.07 | 0.005 | -12.96 * | **0.01** | **0.003** | **3.51 *** |

## Note S4
## Bayes factor-based evidence against DLM as an independent constraint hypothesis

A key result in our paper is that there is no evidence to support DLM as an independent constraint hypothesis when real trees are compared with IC-matched RLAs. But the null result is insufficient to conclude that there is evidence against the DLM as an independent constraint hypothesis. In order to evaluate direct evidence in favor of a null hypothesis against DLM as an independent constraint, we do a Bayes factor analysis. The models and results are shown next.

### Models

Suppose $DL_{ij}$ is the mean intervener complexity for $i^{th}$ sentence of the $j^{th}$ language, $S_{ij}$ is the length of $i^{th}$ sentence of the $j^{th}$ language, $R_{ij}$ is a dummy variable that encodes whether the sentence is a real tree (as 1) or a baseline tree (as 0), $\beta_0$ is the intercept term, $\beta_1$ and $\beta_2$ are the slope terms for the main effect of sentence length and real/baseline variable respectively, $\beta_3$ is the interaction term, $u_{0,j}$ is the random intercept adjustment for $j^{th}$ language, $u_{1,j}$, $u_{2,j}$ and $u_{3,j}$ are random slope adjustments for the $j^{th}$ language.

The **DLM as an independent constraint model** to predict $DL_{ij}$ is shown below

$$DL_{ij} \sim Normal(\beta_0 + u_{0,j}) + (\beta_1 + u_{1,j})S_{ij} + (\beta_2 + u_{2,j})R_{ij} + (\beta_3 + u_{3,j})S_{ij} * R_{ij}, \sigma)$$

Since DLM as an independent constraint model should predict that rate of growth of DL w.r.t. sentence length is slower in real trees compared to IC-matched RLAs, we choose a truncated normal prior on interaction effect parameter, $\beta_3$ such that only negative values are allowed,

$$\beta_3 \sim Normal_{-}(0, \sigma_p)$$

where $\sigma_p \in \{0.0005, 0.001, 0.005, 0.01, 0.05, 0.1, 0.5\}$; the value of $\sigma_p$ expresses our prior belief about the plausible range of true effect size. For example, $\sigma_p = 0.0005$ implies that the interaction effect lies somewhere between 0 and 0.0001. We have chosen a range of reasonable values for $\sigma_p$ because the Bayes factors could be very sensitive to prior on the interaction effect parameter (**?**).

The rationale for choosing the above range of priors is that the interaction effect estimates from other five baselines (except the IC-matched RLAs we have considered here) lie in the range of 0.02 to 0.2. Moreover, in the corpus studies that report the slope of increase in dependency length with respect to sentence length, the difference in slopes of DL-growth for real and random trees is in the range of 0.02 to 0.3 (Ferrer-i Cancho, 2004; Futrell, Mahowald, & Gibson, 2015). Therefore, it is reasonable to believe that if the interaction effect exists it should be somewhere between 0 and 0.5 such that if the true effect size is very small, it could be in the range of 0 to 0.001 (the most conservative prior[2]), and if it is large, it could be in the range of 0 to 0.5. Our priors on the interaction effect express exactly this belief.

[2]We call it the most conservative prior because it is least likely to produce evidence against the DLM as an independent constraint model.

The **Null model**[3] to predict $DL_{ij}$ is shown below,

$$DL_{ij} \sim Normal(\beta_0 + u_{0,j}) + (\beta_1 + u_{1,j})S_{ij} + (\beta_2 + u_{2,j})R_{ij} + (u_{3,j})S_{ij} * R_{ij}, \sigma)$$

We fit the above models using Stan (**??**) and compute Bayes factors from fitted models using bridgesampling (**?**).

## Results

Figure S2 shows the Bayes factors (degree of evidence) in the favour of NULL hypothesis against the DLM as independent constraint hypothesis. We find moderate to strong evidence in the favor of NULL hypothesis – Bayes factors are larger than 5 for all the priors on interaction effect parameter including the most conservative priors, $Normal_{-}(0, .0005)$ and $Normal_{-}(0, 0.001)$). This suggests that our confidence in accepting the NULL hypothesis should be reasonably high.

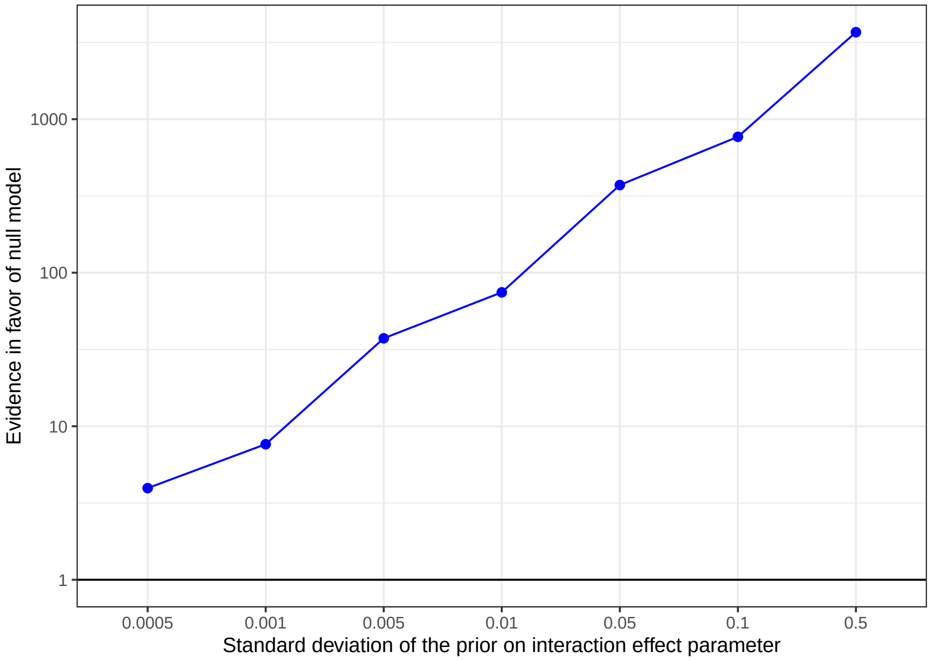


**Figure S2**

*Bayes factor based evidence against DLM as an independent constraint hypothesis: Bayes factors in favour of a null model lacking interaction effect term against a full model with interaction effect. Bayes factors are shown for a range of priors on the interaction effect term. Since DLM as an independent constraint hypothesis predicts negative estimate of interaction effect, we choose truncated normal priors on interaction effect allowing only negative values,* $Normal_{-}(0, \sigma)$*; different values of standard deviation* $\sigma$ *were used to check Bayes factor sensitivity, they are shown on the x-axis.*

[3]Note that the Null model lacks the interaction effect parameter, $\beta_3$

## Note S5
## Analysis using data from clauses taken from sentences longer than 12 words

**Table S2**

*DLM as an independent constraint: estimates from the fitted linear-mixed models for IC-matched random structures and IC-matched RLAs.*

| | IC-matched random structures | | | IC-matched RLAs | | |
|---|---|---|---|---|---|---|
| | **Estimate** | **Std. Error** | **t-value** | **Estimate** | **Std. Error** | **t-value** |
| **Intercept** | 1.906 | 0.018 | 100.88 * | 2.235 | 0.028 | 78.10 * |
| **S.length** | 0.240 | 0.010 | 23.21 * | 0.396 | 0.009 | 42.46 * |
| **Real** | -0.151 | 0.015 | -9.57 * | 0.023 | 0.005 | 4.16 * |
| **S.length:Real** | -0.028 | 0.005 | -4.94 * | 0.008 | 0.002 | 4.04 * |

## Note S6
## Effect of word-order typology on IC/DL minimization: Linear-mixed models with linguistic typology as a predictor

We test whether the constraint on intervener complexity and dependency lengths differ across typologically distinct languages. In particular, we ask whether IC/DL is minimized in both SVO vs SOV languages and whether the rate of minimization differ across typology? We refit the models shown in section 2.3 in the paper with an additional predictor, language typology. For more controlled analyses, we choose only SOV and SVO typology languages.

Since we are interested in the interaction effect of sentence length and real/random variable to test the minimization hypotheses, we add nested contrasts in the model to obtain the interaction effect for SOV and SVO languages.

Let, $IC_{ij}$ be the (average) intervener complexity for sentence $i$ in language $j$. $R_{ij}$ be the dummy variable encoding whether the sentence is from real language or random baseline, $S_{ij}$ be the $i^{th}$ sentence of language $j$ and $T_j$ is the typology of language $j$. $SR_{SVO,ij}$ and $SR_{SOV,ij}$ are nested contrasts encoding interaction of sentence length and real/random variable within SVO and SOV languages respectively. $u_{0j}$ is random intercept for language $j$. $\beta_0$, $\beta_1$, $\beta_2$ and $\beta_3$ are the parameters associated with intercept, effect of sentence length, effect of real/random variable and effect of typology respectively. $\beta_{svo}$ and $\beta_{sov}$ represent interaction effect between sentence length and real/random variable within SVO and SOV languages.

$$IC_{ij} = (\beta_0 + u_{0j}) + \beta_1 S_{ij} + \beta_2 R_{ij} + \beta_3 T_j + \beta_{svo} SR_{SVO,ij} + \beta_{sov} SR_{SOV,ij} + \epsilon \quad (2)$$

We check the estimate of nested interaction effects $\beta_{svo}$ and $\beta_{sov}$ to test the IC/DL minimization hypotheses[4]. If DL/IC is minimized in real trees against baseline trees, the $\hat{\beta_{svo}}$ and $\hat{\beta_{sov}}$ will be negative. $\hat{\beta_{svo}}$ represents to what extent does IC/DL grow slower in SVO language trees compared to baseline trees, while $\hat{\beta_{sov}}$ represents to what extent does IC/DL grow slower in SOV language trees compared to baseline trees. As per our results on aggregated data shown in section 3 in the paper, we expect negative estimate for nested interaction effects for SOV and SVO for all baselines except for IC-matched RLAs.

Table S3 shows the estimates of interaction effect between sentence length and real/random variable within SOV and SVO languages. The significant effects are highlighted in bold

we find that both SOV and SVO languages show expected DL and IC minimization that was found in the aggregated data, i.e., IC/DL grows significantly slower in real trees compared to random baseline trees (except IC matched RLAs). At the same time, the effect of minimization is weaker in SOV language compared to SVO languages suggesting a degree of linguistic adaptability in SOV languages (cf Yadav, Vaidya, Shukla, & Husain, 2020). See Note S6 in the supplementary materials for detailed results. Together these additional analyses suggest that results obtained on the aggregated data can be generalized to these typologically distinct languages.

[4]The dependent variable will be dependency length to test the DL minimization hypotheses using the same model.

**Table S3**

*Interaction between word-order typology and IC/DL minimization: Interaction effect within SOV and SVO languages for various random baselines*

| **Baseline** | **Interaction effect estimate** | |
|---|---|---|
| | **SVO** | **SOV** |
| | $\beta_{svo}$ | $\beta_{sov}$ |
| Random structures | **-0.0954** | **-0.0550** |
| Random linear arrangements | **-0.0680** | **-0.0353** |
| DL-matched random structures | **-0.0202** | **-0.0171** |
| DL-matched RLAs | **-0.0097** | **-0.0053** |
| IC-matched random structures | **-0.0379** | **-0.0090** |
| IC-matched RLAs | **0.0036** | **0.0130** |

## Note S7
## Distribution of intervener complexity in real trees against random structure trees

**Model:** $IC_i = \beta_0 + \beta_1 S_i + \beta_2 R_i + \beta_3 S_i * R_i + \epsilon$,

where $S_i$ is the length of the $i^{th}$ sentence, $R_i$ is the dummy variable encoding whether the $i^{th}$ tree is a real tree or baseline tree. The interaction effect estimate $\hat{\beta}_3$ represents to what extent intervener complexity grows slower w.r.t. sentence length in real trees compared to baseline trees. Intervener Complexity Minimization (ICM) hypothesis predicts negative sign for $\hat{\beta}_3$.

**Results:** Intervener complexity is minimized against random structures baseline in all the languages except *Northern Kurdish.*

**Table S4**

*The table shows the estimate, standard error and t value for the interaction effect coefficient, $\beta_3$ for each language. * signifies significance at p-value < 0.05.*

| | Language | Size | Estimate | Std. Error | t value | |
|---|---|---|---|---|---|---|
| 1 | Afrikaans | 195 | −0.13 | 0.044 | −2.91 | * |
| 2 | Amharic | 877 | −0.17 | 0.016 | −11.00 | * |
| 3 | Arabic | 2063 | −0.18 | 0.007 | −25.03 | * |
| 4 | Armenian | 233 | −0.17 | 0.027 | −6.25 | * |
| 5 | Bambara | 627 | −0.21 | 0.014 | −14.55 | * |
| 6 | Bulgarian | 4764 | −0.20 | 0.006 | −35.61 | * |
| 7 | Catalan | 1527 | −0.22 | 0.011 | −20.16 | * |
| 8 | Chinese | 588 | −0.20 | 0.043 | −4.60 | * |
| 9 | Croatian | 1627 | −0.21 | 0.012 | −16.76 | * |
| 10 | Czech | 26 477 | −0.19 | 0.002 | −86.71 | * |
| 11 | Danish | 1661 | −0.21 | 0.009 | −22.92 | * |
| 12 | Dutch | 6487 | −0.16 | 0.006 | −27.63 | * |
| 13 | English | 4986 | −0.20 | 0.004 | −44.78 | * |
| 14 | Erzya | 1217 | −0.20 | 0.010 | −19.31 | * |
| 15 | Estonian | 13 271 | −0.18 | 0.003 | −53.75 | * |
| 16 | Faroese | 1541 | −0.38 | 0.018 | −21.18 | * |
| 17 | Finnish | 7258 | −0.19 | 0.005 | −41.68 | * |
| 18 | French | 2193 | −0.19 | 0.008 | −23.66 | * |
| 19 | German | 21 446 | −0.14 | 0.003 | −47.23 | * |
| 20 | Greek | 298 | −0.20 | 0.027 | −7.35 | * |
| 21 | Hebrew | 905 | −0.17 | 0.014 | −12.64 | * |
| 22 | Hindi | 2384 | −0.17 | 0.014 | −11.74 | * |
| 23 | Hungarian | 251 | −0.18 | 0.031 | −5.68 | * |
| 24 | Indonesian | 1291 | −0.22 | 0.013 | −16.40 | * |
| 25 | Irish | 183 | −0.16 | 0.026 | −6.22 | * |
| 26 | Italian | 4576 | −0.21 | 0.005 | −38.33 | * |
| 27 | Japanese | 5330 | −0.16 | 0.006 | −27.58 | * |
| 28 | Kazakh | 896 | −0.08 | 0.014 | −5.80 | * |

| | | | | | | |
|---|---|---|---|---|---|---|
| 29 | Korean | 11 974 | −0.09 | 0.005 | −19.22 | * |
| 30 | Latvian | 4459 | −0.20 | 0.006 | −34.26 | * |
| 31 | Lithuanian | 540 | −0.15 | 0.016 | −9.36 | * |
| 32 | Maltese | 379 | −0.20 | 0.015 | −13.24 | * |
| 33 | Northern Kurdish | 414 | −0.03 | 0.036 | −0.96 | n.s. |
| 34 | Northern Sami | 2073 | −0.22 | 0.008 | −25.58 | * |
| 35 | Norwegian | 6890 | −0.21 | 0.004 | −50.39 | * |
| 36 | Persian | 1189 | −0.06 | 0.013 | −4.29 | * |
| 37 | Polish | 9056 | −0.19 | 0.005 | −41.32 | * |
| 38 | Portuguese | 2270 | −0.21 | 0.007 | −29.10 | * |
| 39 | Romanian | 3194 | −0.19 | 0.007 | −26.39 | * |
| 40 | Russian | 1433 | −0.19 | 0.012 | −16.47 | * |
| 41 | Serbian | 735 | −0.20 | 0.019 | −10.55 | * |
| 42 | Slovak | 6715 | −0.20 | 0.004 | −45.49 | * |
| 43 | Slovenian | 2475 | −0.21 | 0.009 | −24.12 | * |
| 44 | Spanish | 2201 | −0.21 | 0.008 | −25.95 | * |
| 45 | Swedish | 1876 | −0.18 | 0.009 | −20.41 | * |
| 46 | Tamil | 170 | −0.14 | 0.042 | −3.40 | * |
| 47 | Thai | 101 | −0.21 | 0.052 | −3.99 | * |
| 48 | Turkish | 2710 | −0.12 | 0.007 | −17.76 | * |
| 49 | Uighur | 1214 | −0.07 | 0.013 | −5.91 | * |
| 50 | Ukrainian | 2703 | −0.19 | 0.007 | −27.22 | * |
| 51 | Upper Sorbian | 283 | −0.15 | 0.036 | −4.19 | * |
| 52 | Urdu | 466 | −0.13 | 0.031 | −4.21 | * |
| 53 | Vietnamese | 622 | −0.21 | 0.017 | −12.52 | * |
| 54 | Wolof | 376 | −0.20 | 0.027 | −7.37 | * |

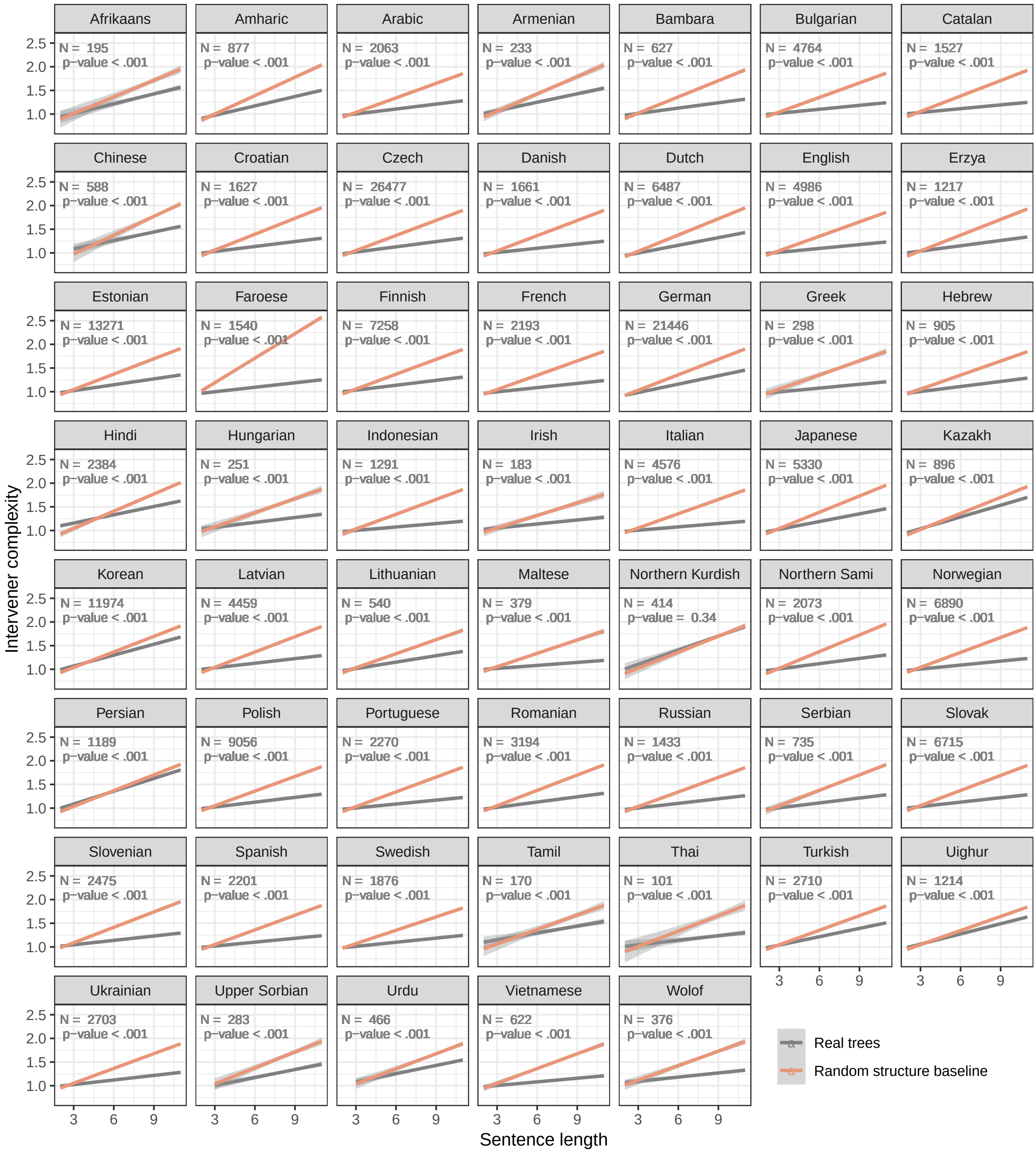


**Figure S3**

*Distribution of intervener complexity in natural language trees against random structure trees. Size of the data, N, and p-value for the interaction effect $\beta_3$ are shown for each language. A p-value of less than .05 represents whether the interaction effect, i.e., $\beta_3$ is significant.*

## Note S8
## Distribution of intervener complexity in real trees against random linear arrangements (RLAs)

**Model:** $IC_i = \beta_0 + \beta_1 S_i + \beta_2 R_i + \beta_3 S_i * R_i + \epsilon$,

where $S_i$ is the length of the $i^{th}$ sentence, $R_i$ is the dummy variable encoding whether the $i^{th}$ tree is a real tree or baseline tree. The interaction effect estimate $\hat{\beta}_3$ represents to what extent intervener complexity grows slower w.r.t. sentence length in real trees compared to baseline trees. Intervener Complexity Minimization (ICM) hypothesis predicts negative sign for $\hat{\beta}_3$.

**Results:** Intervener complexity is minimized against random linear arrangements in all the languages except *Chinese*, *Northern Kurdish*, and *Upper Sorbian.*

**Table S5**

*The table shows the estimate, standard error and t value for the interaction effect coefficient, $\beta_3$ for each language. * signifies significance at p-value < 0.05.*

| | Language | Size | Estimate | Std. Error | t value | |
|---|---|---|---|---|---|---|
| 1 | Afrikaans | 191 | −0.17 | 0.044 | −3.93 | * |
| 2 | Amharic | 763 | −0.08 | 0.017 | −4.54 | * |
| 3 | Arabic | 1680 | −0.16 | 0.010 | −17.06 | * |
| 4 | Armenian | 202 | −0.13 | 0.030 | −4.44 | * |
| 5 | Bambara | 543 | −0.16 | 0.017 | −9.58 | * |
| 6 | Bulgarian | 4294 | −0.17 | 0.006 | −26.37 | * |
| 7 | Catalan | 1419 | −0.17 | 0.011 | −14.90 | * |
| 8 | Chinese | 588 | −0.07 | 0.036 | −1.92 | n.s. |
| 9 | Croatian | 1547 | −0.14 | 0.012 | −11.08 | * |
| 10 | Czech | 22520 | −0.13 | 0.003 | −52.19 | * |
| 11 | Danish | 1429 | −0.26 | 0.012 | −22.41 | * |
| 12 | Dutch | 5905 | −0.11 | 0.006 | −18.24 | * |
| 13 | English | 4040 | −0.16 | 0.006 | −27.77 | * |
| 14 | Erzya | 965 | −0.12 | 0.012 | −9.95 | * |
| 15 | Estonian | 11182 | −0.11 | 0.004 | −29.42 | * |
| 16 | Faroese | 1004 | −0.16 | 0.014 | −11.94 | * |
| 17 | Finnish | 6312 | −0.12 | 0.005 | −23.81 | * |
| 18 | French | 1993 | −0.13 | 0.009 | −15.69 | * |
| 19 | German | 19516 | −0.05 | 0.003 | −17.88 | * |
| 20 | Greek | 290 | −0.20 | 0.026 | −7.84 | * |
| 21 | Hebrew | 850 | −0.12 | 0.014 | −8.79 | * |
| 22 | Hindi | 2348 | −0.14 | 0.014 | −10.26 | * |
| 23 | Hungarian | 244 | −0.08 | 0.025 | −3.35 | * |
| 24 | Indonesian | 1229 | −0.15 | 0.013 | −11.47 | * |
| 25 | Irish | 167 | −0.14 | 0.026 | −5.20 | * |
| 26 | Italian | 4099 | −0.15 | 0.006 | −24.38 | * |
| 27 | Japanese | 10644 | −0.14 | 0.005 | −28.59 | * |
| 28 | Kazakh | 788 | −0.04 | 0.015 | −2.75 | * |

| | | | | | | |
|---|---|---|---|---|---|---|
| 29 | Korean | 11181 | −0.06 | 0.005 | −13.87 | * |
| 30 | Latvian | 3910 | −0.14 | 0.006 | −21.09 | * |
| 31 | Lithuanian | 474 | −0.10 | 0.016 | −6.36 | * |
| 32 | Maltese | 280 | −0.17 | 0.020 | −8.66 | * |
| 33 | Northern Kurdish | 404 | −0.03 | 0.036 | −0.91 | n.s. |
| 34 | Northern Sami | 1534 | −0.12 | 0.010 | −12.02 | * |
| 35 | Norwegian | 5678 | −0.16 | 0.005 | −30.23 | * |
| 36 | Persian | 1071 | −0.05 | 0.014 | −3.83 | * |
| 37 | Polish | 8366 | −0.14 | 0.005 | −29.98 | * |
| 38 | Portuguese | 1992 | −0.15 | 0.008 | −18.75 | * |
| 39 | Romanian | 2944 | −0.12 | 0.007 | −16.66 | * |
| 40 | Russian | 1399 | −0.14 | 0.010 | −13.66 | * |
| 41 | Serbian | 711 | −0.12 | 0.019 | −6.68 | * |
| 42 | Slovak | 5335 | −0.14 | 0.005 | −26.52 | * |
| 43 | Slovenian | 2217 | −0.17 | 0.010 | −17.32 | * |
| 44 | Spanish | 1956 | −0.14 | 0.008 | −16.86 | * |
| 45 | Swedish | 1683 | −0.14 | 0.010 | −13.84 | * |
| 46 | Tamil | 166 | −0.11 | 0.040 | −2.81 | * |
| 47 | Thai | 97 | −0.13 | 0.045 | −2.87 | * |
| 48 | Turkish | 2147 | −0.08 | 0.008 | −10.55 | * |
| 49 | Uighur | 1083 | −0.03 | 0.013 | −2.30 | * |
| 50 | Ukrainian | 2270 | −0.13 | 0.008 | −16.20 | * |
| 51 | Upper Sorbian | 276 | −0.03 | 0.030 | −1.15 | n.s. |
| 52 | Urdu | 461 | −0.13 | 0.030 | −4.40 | * |
| 53 | Vietnamese | 568 | −0.15 | 0.016 | −9.54 | * |
| 54 | Wolof | 359 | −0.22 | 0.028 | −7.85 | * |

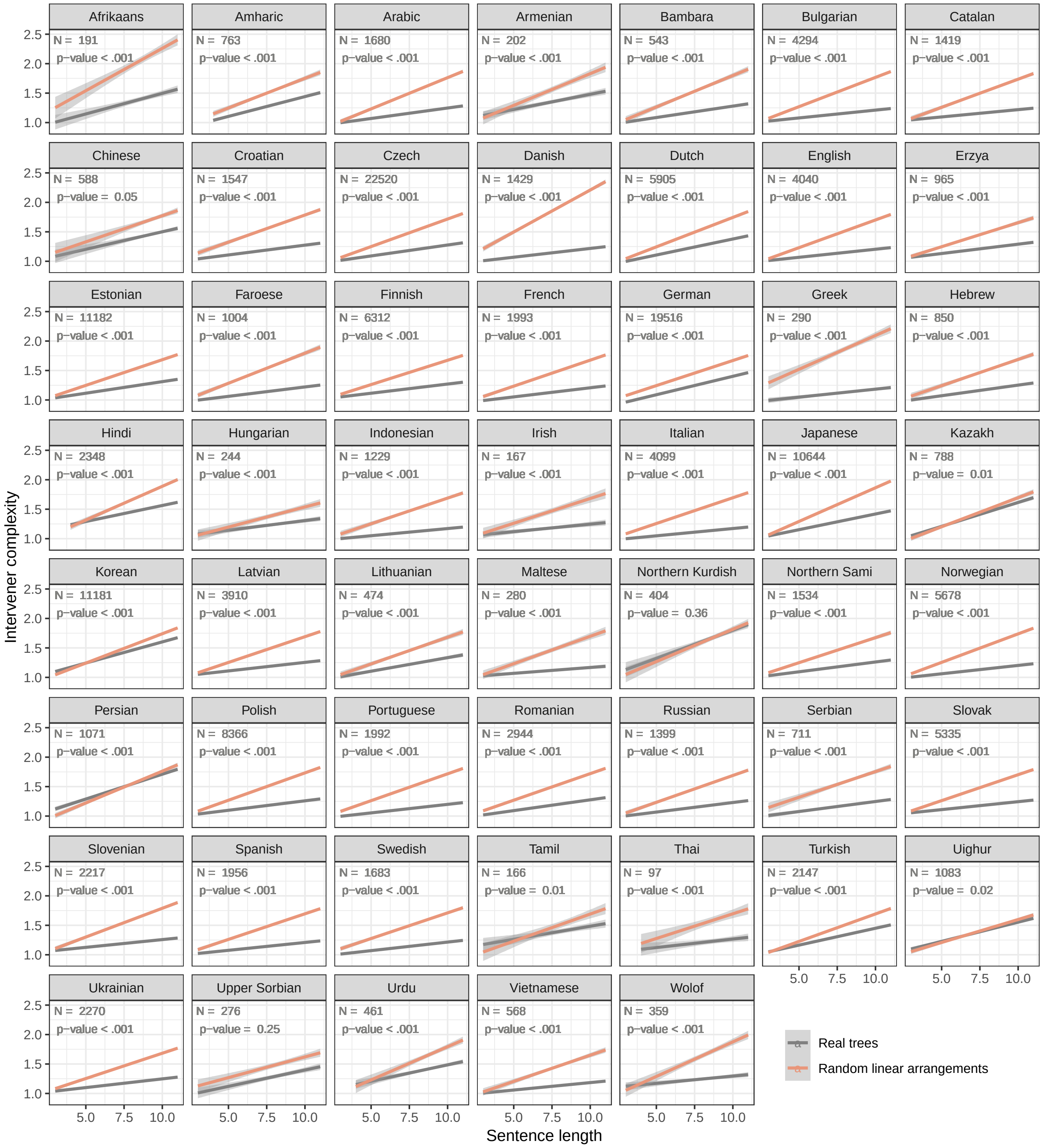


**Figure S4**

*Distribution of intervener complexity in natural language trees against RLAs. Size of the data, N, and p-value for the interaction effect $\beta_3$ are shown for each language. A p-value of less than .05 represents whether the interaction effect, i.e., $\beta_3$ is significant.*

## Note S9
## Distribution of intervener complexity in real trees against DL-matched random structures

**Model:** $IC_i = \beta_0 + \beta_1 S_i + \beta_2 R_i + \beta_3 S_i * R_i + \epsilon$,

where $S_i$ is the length of the $i^{th}$ sentence, $R_i$ is the dummy variable encoding whether the $i^{th}$ tree is a real tree or baseline tree. The interaction effect estimate $\hat{\beta}_3$ represents to what extent intervener complexity grows slower w.r.t. sentence length in real trees compared to baseline trees. Intervener Complexity Minimization (ICM) hypothesis predicts negative sign for $\hat{\beta}_3$.

**Results:** Intervener complexity is minimized against DL-matched random structures in the languages where sufficient sample size is available (i.e., when sentence count is $> 1000$), except *Arabic* and *Turkish.*

**Table S6**

*The table shows the estimate, standard error and t value for the interaction effect coefficient, $\beta_3$ for each language. * signifies significance at p-value* $< 0.05$.

| | Language | Size | Estimate | Std. Error | t value | |
|---|---|---|---|---|---|---|
| 1 | Afrikaans | 158 | −0.20 | 0.044 | −4.46 | * |
| 2 | Amharic | 614 | −0.02 | 0.015 | −1.54 | n.s. |
| 3 | Arabic | 1446 | −0.01 | 0.006 | −1.45 | n.s. |
| 4 | Armenian | 178 | −0.01 | 0.022 | −0.55 | n.s. |
| 5 | Bambara | 442 | −0.01 | 0.014 | −1.02 | n.s. |
| 6 | Bulgarian | 3443 | −0.01 | 0.005 | −2.74 | * |
| 7 | Catalan | 991 | −0.02 | 0.009 | −1.95 | n.s. |
| 8 | Chinese | 314 | −0.02 | 0.039 | −0.55 | n.s. |
| 9 | Croatian | 1006 | −0.03 | 0.010 | −3.07 | * |
| 10 | Czech | 18 819 | −0.02 | 0.002 | −12.98 | * |
| 11 | Danish | 1220 | −0.08 | 0.008 | −11.03 | * |
| 12 | Dutch | 5121 | −0.05 | 0.005 | −8.73 | * |
| 13 | English | 3713 | −0.03 | 0.003 | −8.79 | * |
| 14 | Erzya | 951 | −0.02 | 0.010 | −1.54 | n.s. |
| 15 | Estonian | 10 475 | −0.09 | 0.003 | −29.47 | * |
| 16 | Faroese | 864 | −0.02 | 0.011 | −1.88 | n.s. |
| 17 | Finnish | 4913 | −0.02 | 0.004 | −4.83 | * |
| 18 | French | 1553 | −0.05 | 0.006 | −7.98 | * |
| 19 | German | 12 763 | −0.03 | 0.003 | −9.57 | * |
| 20 | Greek | 181 | 0.00 | 0.019 | −0.25 | n.s. |
| 21 | Hebrew | 526 | 0.00 | 0.011 | −0.32 | n.s. |
| 22 | Hindi | 1450 | −0.13 | 0.013 | −10.14 | * |
| 23 | Hungarian | 160 | −0.03 | 0.025 | −1.20 | n.s. |
| 24 | Indonesian | 660 | −0.02 | 0.011 | −1.40 | n.s. |
| 25 | Irish | 129 | −0.02 | 0.027 | −0.73 | n.s. |
| 26 | Italian | 3147 | −0.05 | 0.004 | −11.26 | * |
| 27 | Japanese | 3291 | −0.02 | 0.007 | −3.52 | * |

| | | | | | | |
|---|---|---|---|---|---|---|
| 28 | Kazakh | 685 | 0.00 | 0.014 | −0.16 | n.s. |
| 29 | Korean | 6207 | 0.00 | 0.005 | −0.98 | n.s. |
| 30 | Latvian | 2794 | −0.02 | 0.006 | −3.31 | * |
| 31 | Lithuanian | 381 | −0.01 | 0.014 | −1.06 | n.s. |
| 32 | Maltese | 281 | −0.01 | 0.011 | −1.21 | n.s. |
| 33 | Northern Kurdish | 178 | −0.01 | 0.045 | −0.17 | n.s. |
| 34 | Northern Sami | 1858 | −0.04 | 0.007 | −5.99 | * |
| 35 | Norwegian | 4759 | −0.02 | 0.003 | −6.11 | * |
| 36 | Persian | 827 | 0.00 | 0.013 | 0.35 | n.s. |
| 37 | Polish | 5218 | 0.00 | 0.005 | −0.29 | n.s. |
| 38 | Portuguese | 1632 | −0.05 | 0.006 | −8.89 | * |
| 39 | Romanian | 1903 | −0.03 | 0.008 | −4.11 | * |
| 40 | Russian | 838 | 0.00 | 0.009 | 0.39 | n.s. |
| 41 | Serbian | 399 | −0.02 | 0.015 | −1.53 | n.s. |
| 42 | Slovak | 5503 | −0.03 | 0.004 | −7.01 | * |
| 43 | Slovenian | 1962 | −0.14 | 0.008 | −18.05 | * |
| 44 | Spanish | 1276 | −0.01 | 0.007 | −1.45 | n.s. |
| 45 | Swedish | 1212 | −0.02 | 0.007 | −2.82 | * |
| 46 | Tamil | 108 | 0.00 | 0.037 | 0.09 | n.s. |
| 47 | Thai | 42 | −0.01 | 0.049 | −0.21 | n.s. |
| 48 | Turkish | 2286 | −0.01 | 0.006 | −1.36 | n.s. |
| 49 | Uighur | 878 | −0.01 | 0.012 | −0.63 | n.s. |
| 50 | Ukrainian | 1915 | −0.03 | 0.006 | −4.34 | * |
| 51 | Upper Sorbian | 142 | −0.04 | 0.042 | −0.95 | n.s. |
| 52 | Urdu | 168 | −0.01 | 0.046 | −0.28 | n.s. |
| 53 | Vietnamese | 399 | −0.05 | 0.012 | −4.33 | * |
| 54 | Wolof | 241 | −0.03 | 0.022 | −1.48 | n.s. |

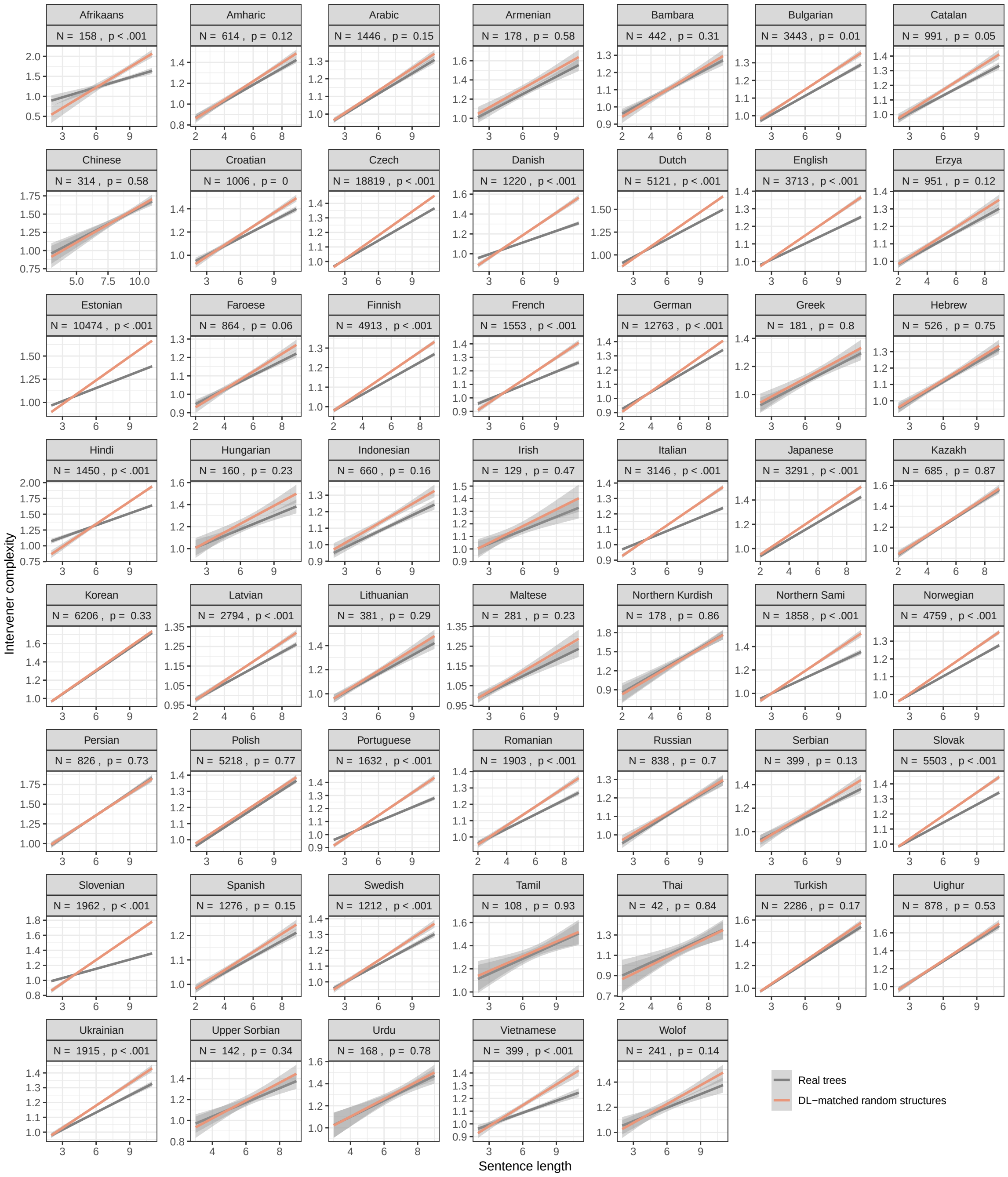


**Figure S5**

*Distribution of intervener complexity in natural language trees against DL-matched random structures. Size of the data, N, and p-value for the interaction effect $\beta_3$ are shown for each language. A p-value of less than .05 represents whether the interaction effect, i.e., $\beta_3$ is significant.*

## Note S10
## Distribution of intervener complexity in real trees against DL-matched RLAs

**Model:** $IC_i = \beta_0 + \beta_1 S_i + \beta_2 R_i + \beta_3 S_i * R_i + \epsilon$,

where $S_i$ is the length of the $i^{th}$ sentence, $R_i$ is the dummy variable encoding whether the $i^{th}$ tree is a real tree or baseline tree. The interaction effect estimate $\hat{\beta}_3$ represents to what extent intervener complexity grows slower w.r.t. sentence length in real trees compared to baseline trees. Intervener Complexity Minimization (ICM) hypothesis predicts negative sign for $\hat{\beta}_3$.

**Results:** DL-matched random linear arrangements show mixed results. The intervener complexity is minimized against DL-matched RLAs in *Afrikaans, Czech, Danish, Dutch, English, Estonian, German, Greek, Hindi, Korean, Norwegian, Portuguese, Slovak,* and *Slovenian.* Most of the languages which show significant effect have relatively large sample size. The possible reason for mixed results across languages is that the effect size is quite small and cannot be detected in languages with a small treebank size.

**Table S7**

*The table shows the estimate, standard error and t value for the interaction effect coefficient, $\beta_3$ for each language. * signifies significance at p-value $< 0.05$.*

| | Language | Size | Estimate | Std. Error | t value | |
|---|---|---|---|---|---|---|
| 1 | Afrikaans | 165 | −0.15 | 0.043 | −3.50 | * |
| 2 | Amharic | 483 | −0.01 | 0.022 | −0.53 | n.s. |
| 3 | Arabic | 1423 | 0.00 | 0.006 | 0.42 | n.s. |
| 4 | Armenian | 181 | −0.01 | 0.024 | −0.28 | n.s. |
| 5 | Bambara | 339 | −0.01 | 0.020 | −0.33 | n.s. |
| 6 | Bulgarian | 3733 | −0.01 | 0.005 | −1.48 | n.s. |
| 7 | Catalan | 1187 | 0.00 | 0.008 | −0.36 | n.s. |
| 8 | Chinese | 466 | −0.01 | 0.031 | −0.28 | n.s. |
| 9 | Croatian | 1333 | −0.01 | 0.009 | −1.49 | n.s. |
| 10 | Czech | 19 516 | −0.01 | 0.002 | −6.41 | * |
| 11 | Danish | 1301 | −0.09 | 0.009 | −9.58 | * |
| 12 | Dutch | 5415 | −0.02 | 0.005 | −3.58 | * |
| 13 | English | 3550 | −0.01 | 0.004 | −2.02 | * |
| 14 | Erzya | 696 | −0.02 | 0.015 | −1.04 | n.s. |
| 15 | Estonian | 9959 | −0.01 | 0.003 | −4.11 | * |
| 16 | Faroese | 730 | −0.02 | 0.015 | −1.10 | n.s. |
| 17 | Finnish | 5580 | −0.01 | 0.004 | −1.67 | n.s. |
| 18 | French | 1673 | −0.01 | 0.006 | −0.97 | n.s. |
| 19 | German | 10 436 | −0.02 | 0.004 | −5.25 | * |
| 20 | Greek | 264 | −0.07 | 0.019 | −3.98 | * |
| 21 | Hebrew | 658 | −0.01 | 0.011 | −0.53 | n.s. |
| 22 | Hindi | 1818 | −0.03 | 0.010 | −2.64 | * |
| 23 | Hungarian | 221 | 0.00 | 0.019 | −0.04 | n.s. |
| 24 | Indonesian | 990 | −0.01 | 0.009 | −0.90 | n.s. |
| 25 | Irish | 143 | 0.01 | 0.021 | 0.39 | n.s. |

| | | | | | | |
|---|---|---|---|---|---|---|
| 26 | Italian | 3466 | 0.00 | 0.004 | −0.91 | n.s. |
| 27 | Japanese | 4921 | 0.00 | 0.006 | 0.18 | n.s. |
| 28 | Kazakh | 591 | 0.01 | 0.019 | 0.50 | n.s. |
| 29 | Korean | 9793 | −0.03 | 0.004 | −6.72 | * |
| 30 | Latvian | 3427 | −0.01 | 0.005 | −1.05 | n.s. |
| 31 | Lithuanian | 421 | −0.01 | 0.014 | −0.51 | n.s. |
| 32 | Maltese | 237 | 0.00 | 0.013 | 0.08 | n.s. |
| 33 | Northern Kurdish | 158 | −0.02 | 0.071 | −0.31 | n.s. |
| 34 | Northern Sami | 1468 | −0.01 | 0.008 | −1.78 | n.s. |
| 35 | Norwegian | 5154 | −0.07 | 0.004 | −16.39 | * |
| 36 | Persian | 909 | −0.01 | 0.014 | −0.70 | n.s. |
| 37 | Polish | 7167 | 0.00 | 0.003 | −0.36 | n.s. |
| 38 | Portuguese | 1752 | −0.05 | 0.006 | −9.27 | * |
| 39 | Romanian | 2594 | −0.01 | 0.006 | −1.27 | n.s. |
| 40 | Russian | 1117 | −0.01 | 0.008 | −0.90 | n.s. |
| 41 | Serbian | 569 | −0.01 | 0.013 | −0.49 | n.s. |
| 42 | Slovak | 5027 | −0.07 | 0.004 | −16.33 | * |
| 43 | Slovenian | 2008 | −0.10 | 0.008 | −12.07 | * |
| 44 | Spanish | 1628 | −0.01 | 0.006 | −1.29 | n.s. |
| 45 | Swedish | 1436 | −0.01 | 0.007 | −1.45 | n.s. |
| 46 | Tamil | 142 | −0.01 | 0.032 | −0.34 | n.s. |
| 47 | Thai | 40 | −0.04 | 0.073 | −0.48 | n.s. |
| 48 | Turkish | 2012 | 0.00 | 0.007 | 0.46 | n.s. |
| 49 | Uighur | 989 | −0.01 | 0.012 | −0.68 | n.s. |
| 50 | Ukrainian | 1994 | −0.01 | 0.006 | −0.94 | n.s. |
| 51 | Upper Sorbian | 134 | −0.01 | 0.051 | −0.13 | n.s. |
| 52 | Urdu | 354 | 0.00 | 0.026 | 0.15 | n.s. |
| 53 | Vietnamese | 475 | −0.01 | 0.010 | −0.85 | n.s. |
| 54 | Wolof | 300 | −0.02 | 0.023 | −0.75 | n.s. |

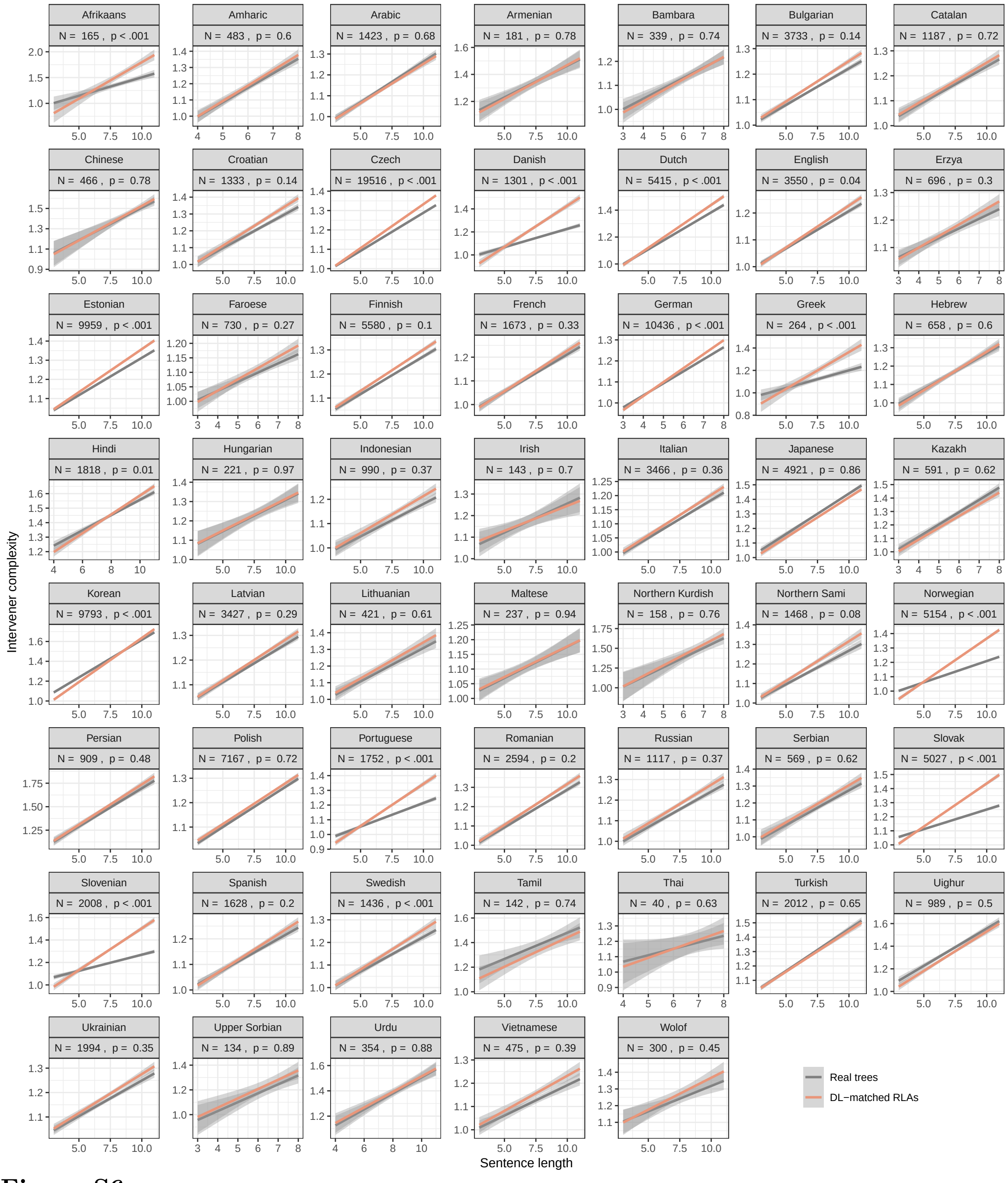


**Figure S6**

*Distribution of intervener complexity in natural language trees against DL-matched RLAs. Size of the data, N, and p-value for the interaction effect* $\beta_3$ *are shown for each language. A p-value of less than .05 represents whether the interaction effect, i.e.,* $\beta_3$ *is significant.*

## Note S11
## Distribution of dependency length in real trees against IC-matched random structures

**Model:** $DL_i = \beta_0 + \beta_1 S_i + \beta_2 R_i + \beta_3 S_i * R_i + \epsilon$,

where $S_i$ is the length of the $i^{th}$ sentence, $R_i$ is the dummy variable encoding whether the $i^{th}$ tree is a real tree or baseline tree. The interaction effect estimate $\hat{\beta}_3$ represents to what extent dependency length grows slower w.r.t. sentence length in real trees compared to baseline trees. Dependency Length Minimization (DLM) as an Independent constraint hypothesis predicts negative sign for $\hat{\beta}_3$.

**Results:** Dependency length is minimized against IC-matched random structures in most of the languages. Interestingly, Dutch does not show dependency length minimization despite a large sample size, suggesting that the dependency length distribution in Dutch is a consequence of constraint on intervener complexity.

**Table S8**

*The table shows the estimate, standard error and t value for the interaction effect coefficient, $\beta_3$ for each language. * signifies significance at p-value $< 0.05$.*

| | Language | Size | Estimate | Std. Error | t value | |
|---|---|---|---|---|---|---|
| 1 | Afrikaans | 175 | 0.00 | 0.026 | −0.15 | n.s. |
| 2 | Amharic | 771 | 0.00 | 0.009 | 0.43 | n.s. |
| 3 | Arabic | 1922 | −0.05 | 0.005 | −9.43 | * |
| 4 | Armenian | 147 | −0.05 | 0.021 | −2.56 | * |
| 5 | Bambara | 522 | −0.04 | 0.010 | −3.47 | * |
| 6 | Bulgarian | 4513 | −0.03 | 0.004 | −9.44 | * |
| 7 | Catalan | 1416 | −0.04 | 0.006 | −6.23 | * |
| 8 | Chinese | 150 | −0.02 | 0.075 | −0.30 | n.s. |
| 9 | Croatian | 860 | −0.03 | 0.011 | −3.07 | * |
| 10 | Czech | 20 311 | −0.03 | 0.002 | −18.81 | * |
| 11 | Danish | 1543 | −0.01 | 0.005 | −1.88 | n.s. |
| 12 | Dutch | 6200 | 0.00 | 0.004 | 0.48 | n.s. |
| 13 | English | 4693 | −0.03 | 0.003 | −11.82 | * |
| 14 | Erzya | 1080 | −0.03 | 0.007 | −4.51 | * |
| 15 | Estonian | 12 641 | −0.02 | 0.002 | −10.64 | * |
| 16 | Faroese | 990 | −0.02 | 0.009 | −1.85 | n.s. |
| 17 | Finnish | 6884 | −0.03 | 0.003 | −10.12 | * |
| 18 | French | 2024 | −0.02 | 0.005 | −5.29 | * |
| 19 | German | 17 944 | 0.01 | 0.002 | 7.02 | * |
| 20 | Greek | 271 | −0.04 | 0.015 | −2.53 | * |
| 21 | Hebrew | 508 | −0.06 | 0.014 | −4.10 | * |
| 22 | Hindi | 2068 | −0.04 | 0.007 | −5.39 | * |
| 23 | Hungarian | 134 | −0.02 | 0.030 | −0.70 | n.s. |
| 24 | Indonesian | 1163 | −0.03 | 0.008 | −4.27 | * |
| 25 | Irish | 129 | −0.05 | 0.026 | −1.85 | n.s. |
| 26 | Italian | 4268 | −0.03 | 0.003 | −9.21 | * |

| | | | | | | |
|---|---|---|---|---|---|---|
| 27 | Japanese | 7052 | −0.03 | 0.003 | −8.93 | * |
| 28 | Kazakh | 768 | −0.02 | 0.010 | −2.14 | * |
| 29 | Korean | 10 624 | −0.02 | 0.003 | −9.26 | * |
| 30 | Latvian | 4197 | −0.04 | 0.003 | −10.25 | * |
| 31 | Lithuanian | 368 | −0.05 | 0.014 | −3.76 | * |
| 32 | Maltese | 282 | −0.05 | 0.014 | −3.45 | * |
| 33 | Northern Kurdish | 251 | 0.00 | 0.023 | 0.02 | n.s. |
| 34 | Northern Sami | 1942 | −0.01 | 0.006 | −0.90 | n.s. |
| 35 | Norwegian | 6447 | −0.02 | 0.002 | −10.04 | * |
| 36 | Persian | 721 | −0.02 | 0.011 | −1.78 | n.s. |
| 37 | Polish | 8484 | −0.04 | 0.003 | −15.87 | * |
| 38 | Portuguese | 2110 | −0.04 | 0.004 | −8.57 | * |
| 39 | Romanian | 3014 | −0.02 | 0.004 | −3.88 | * |
| 40 | Russian | 807 | −0.03 | 0.011 | −2.91 | * |
| 41 | Serbian | 353 | −0.02 | 0.018 | −1.35 | n.s. |
| 42 | Slovak | 6523 | −0.03 | 0.003 | −11.13 | * |
| 43 | Slovenian | 2312 | −0.03 | 0.005 | −5.98 | * |
| 44 | Spanish | 2039 | −0.03 | 0.004 | −8.06 | * |
| 45 | Swedish | 1154 | −0.02 | 0.008 | −3.12 | * |
| 46 | Tamil | 89 | −0.04 | 0.041 | −0.91 | n.s. |
| 47 | Thai | 61 | 0.01 | 0.038 | 0.22 | n.s. |
| 48 | Turkish | 2254 | −0.05 | 0.006 | −7.86 | * |
| 49 | Uighur | 831 | −0.05 | 0.012 | −4.03 | * |
| 50 | Ukrainian | 1842 | −0.04 | 0.007 | −5.33 | * |
| 51 | Upper Sorbian | 190 | −0.03 | 0.026 | −1.25 | n.s. |
| 52 | Urdu | 174 | 0.01 | 0.034 | 0.19 | n.s. |
| 53 | Vietnamese | 389 | −0.02 | 0.015 | −1.17 | n.s. |
| 54 | Wolof | 208 | −0.05 | 0.024 | −2.22 | * |

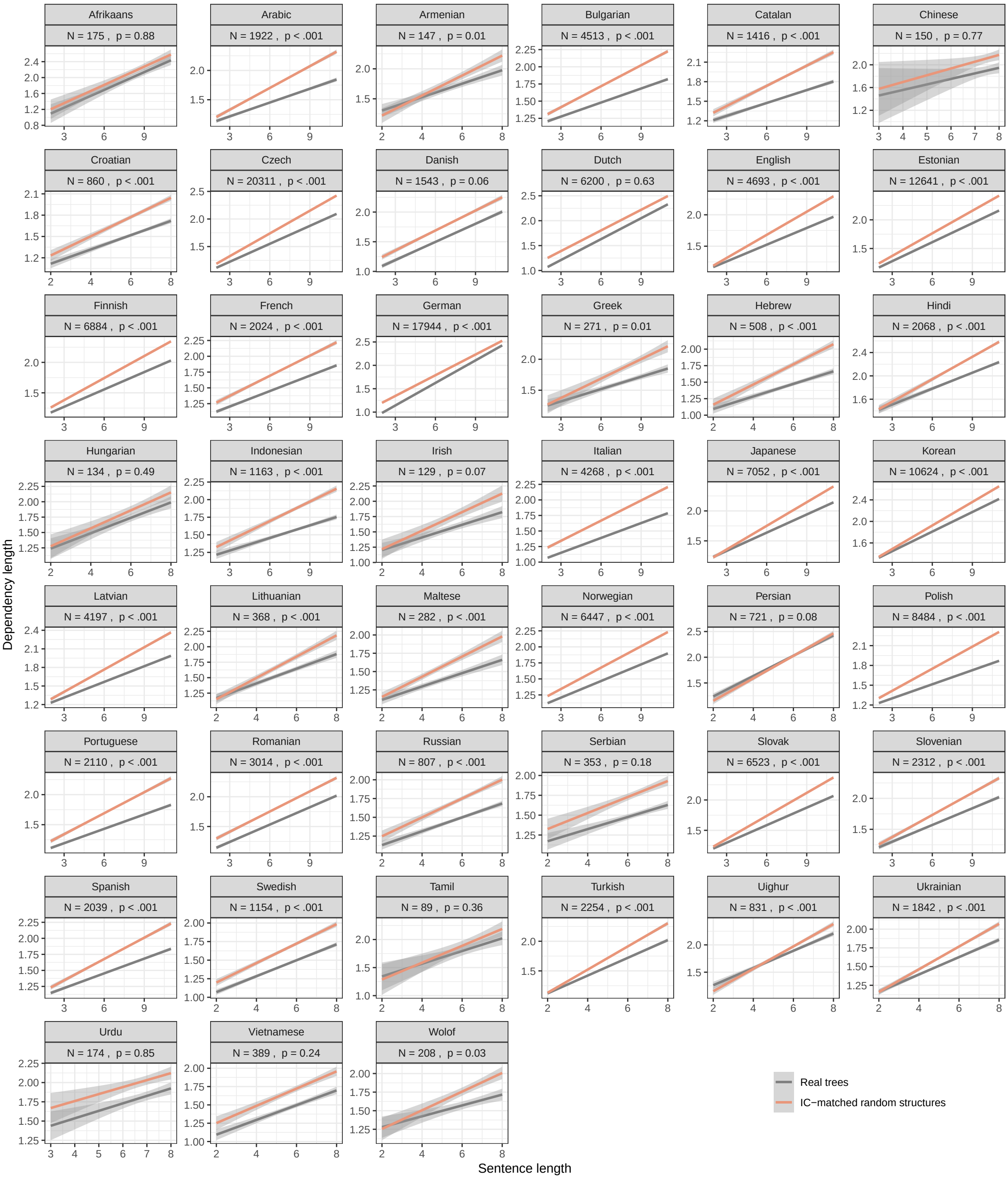


**Figure S7**

*Distribution of dependency length in natural language trees against IC-matched random structures. Size of the data, N, and p-value for the interaction effect $\beta_3$ are shown for each language. A p-value of less than .05 represents whether the interaction effect, i.e., $\beta_3$ is significant.*

## Note S12
## Distribution of dependency length in real trees against IC-matched RLAs

**Model:** $DL_i = \beta_0 + \beta_1 S_i + \beta_2 R_i + \beta_3 S_i * R_i + \epsilon$,

where $S_i$ is the length of the $i^{th}$ sentence, $R_i$ is the dummy variable encoding whether the $i^{th}$ tree is a real tree or baseline tree. The interaction effect estimate $\hat{\beta}_3$ represents to what extent dependency length grows slower w.r.t. sentence length in real trees compared to baseline trees. DLM as an Independent constraint hypothesis predicts negative sign for $\hat{\beta}_3$.

**Results:** None of the languages shows dependency length minimization against IC-matched RLAs. Note that whenever the interaction effect is significant, the direction of the effect is *positive*, which does not support the DLM hypothesis. This result shows that there is no evidence for dependency length minimization cross-linguistically against a baseline matched in intervener complexity distribution and topological structure of the trees.

**Table S9**

*The table shows the estimate, standard error and t value for the interaction effect coefficient, $\beta_3$ for each language. * signifies significance at p-value* $< 0.05$.

| | Language | Size | Estimate | Std. Error | t value | |
|---|---|---|---|---|---|---|
| 1 | Afrikaans | 182 | 0.02 | 0.027 | 0.57 | n.s. |
| 2 | Amharic | 666 | 0.00 | 0.011 | 0.26 | n.s. |
| 3 | Arabic | 1596 | 0.01 | 0.006 | 1.06 | n.s. |
| 4 | Armenian | 114 | −0.03 | 0.030 | −1.09 | n.s. |
| 5 | Bambara | 399 | −0.01 | 0.014 | −0.92 | n.s. |
| 6 | Bulgarian | 4045 | 0.00 | 0.004 | 1.00 | n.s. |
| 7 | Catalan | 1332 | 0.00 | 0.006 | −0.34 | n.s. |
| 8 | Chinese | 149 | 0.02 | 0.077 | 0.21 | n.s. |
| 9 | Croatian | 767 | 0.00 | 0.014 | 0.32 | n.s. |
| 10 | Czech | 21 162 | 0.01 | 0.002 | 4.90 | * |
| 11 | Danish | 1365 | 0.01 | 0.006 | 1.97 | * |
| 12 | Dutch | 5669 | 0.02 | 0.004 | 4.47 | * |
| 13 | English | 3873 | 0.00 | 0.004 | −0.92 | n.s. |
| 14 | Erzya | 814 | −0.01 | 0.009 | −0.90 | n.s. |
| 15 | Estonian | 10 662 | 0.00 | 0.002 | 1.96 | * |
| 16 | Faroese | 792 | 0.01 | 0.011 | 0.96 | n.s. |
| 17 | Finnish | 5993 | 0.00 | 0.003 | −0.13 | n.s. |
| 18 | French | 1867 | 0.00 | 0.005 | 0.89 | n.s. |
| 19 | German | 18 529 | 0.03 | 0.002 | 13.67 | * |
| 20 | Greek | 273 | −0.01 | 0.013 | −0.42 | n.s. |
| 21 | Hebrew | 430 | 0.03 | 0.016 | 1.79 | n.s. |
| 22 | Hindi | 2176 | 0.00 | 0.007 | 0.03 | n.s. |
| 23 | Hungarian | 124 | −0.04 | 0.033 | −1.13 | n.s. |
| 24 | Indonesian | 1139 | 0.01 | 0.008 | 0.88 | n.s. |
| 25 | Irish | 112 | 0.00 | 0.030 | 0.09 | n.s. |
| 26 | Italian | 3796 | 0.01 | 0.003 | 3.42 | * |

| | | | | | | |
|---|---|---|---|---|---|---|
| 27 | Japanese | 6066 | 0.00 | 0.004 | −0.23 | n.s. |
| 28 | Kazakh | 657 | 0.02 | 0.013 | 1.24 | n.s. |
| 29 | Korean | 10 626 | 0.00 | 0.003 | 0.11 | n.s. |
| 30 | Latvian | 3721 | 0.00 | 0.004 | 0.23 | n.s. |
| 31 | Lithuanian | 303 | −0.02 | 0.018 | −1.23 | n.s. |
| 32 | Maltese | 180 | 0.00 | 0.022 | −0.11 | n.s. |
| 33 | Northern Kurdish | 235 | 0.01 | 0.029 | 0.37 | n.s. |
| 34 | Northern Sami | 1383 | −0.01 | 0.007 | −1.27 | n.s. |
| 35 | Norwegian | 5388 | 0.01 | 0.003 | 3.04 | * |
| 36 | Persian | 618 | 0.00 | 0.017 | −0.13 | n.s. |
| 37 | Polish | 7827 | 0.00 | 0.003 | −0.11 | n.s. |
| 38 | Portuguese | 1843 | $9.72 \times 10^{-5}$ | 0.005 | 0.02 | n.s. |
| 39 | Romanian | 2829 | 0.00 | 0.005 | −1.07 | n.s. |
| 40 | Russian | 724 | 0.01 | 0.011 | 1.11 | n.s. |
| 41 | Serbian | 321 | 0.00 | 0.020 | 0.15 | n.s. |
| 42 | Slovak | 5119 | 0.00 | 0.003 | 0.47 | n.s. |
| 43 | Slovenian | 2088 | 0.01 | 0.006 | 0.94 | n.s. |
| 44 | Spanish | 1828 | 0.00 | 0.005 | −0.39 | n.s. |
| 45 | Swedish | 932 | 0.03 | 0.010 | 2.77 | * |
| 46 | Tamil | 86 | −0.02 | 0.050 | −0.46 | n.s. |
| 47 | Thai | 55 | 0.03 | 0.037 | 0.69 | n.s. |
| 48 | Turkish | 1686 | −0.01 | 0.009 | −1.53 | n.s. |
| 49 | Uighur | 715 | 0.01 | 0.016 | 0.79 | n.s. |
| 50 | Ukrainian | 1358 | 0.00 | 0.010 | −0.36 | n.s. |
| 51 | Upper Sorbian | 170 | 0.02 | 0.028 | 0.81 | n.s. |
| 52 | Urdu | 174 | 0.01 | 0.039 | 0.25 | n.s. |
| 53 | Vietnamese | 316 | 0.00 | 0.018 | 0.08 | n.s. |
| 54 | Wolof | 187 | 0.01 | 0.032 | 0.25 | n.s. |

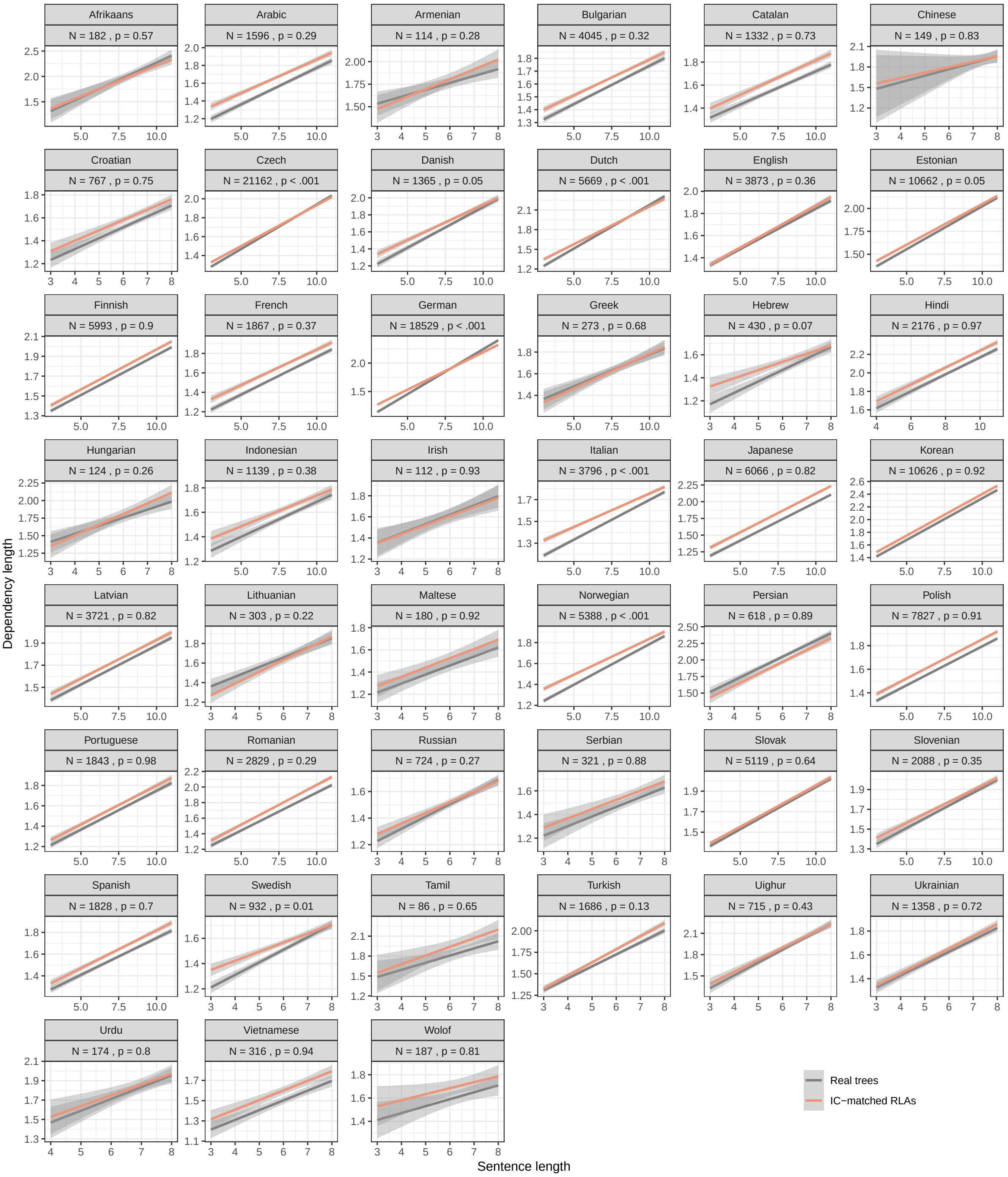


**Figure S8**

*Distribution of dependency length in natural language trees against IC-matched RLAs. Size of the data, N, and p-value for the interaction effect $\beta_3$ are shown for each language. A p-value of less than .05 represents whether the interaction effect, i.e., $\beta_3$, is significant.*

## Note S13
## Analysis using Universal Dependencies treebanks data

### Table S10

*ICM hypothesis: estimates from the fitted linear-mixed models for random structures baseline and random linear arrangements.*

| | Random structures baseline | | | Random linear arrangements | | |
|---|---|---|---|---|---|---|
| | **Estimate** | **Std. Error** | **t-value** | **Estimate** | **Std. Error** | **t-value** |
| **Intercept** | 1.44 | 0.001 | 959.74 * | 1.33 | 0.007 | 184.47 * |
| **S.length** | 0.27 | 0.001 | 262.94 * | 0.14 | 0.004 | 36.04 * |
| **Real** | -0.31 | 0.008 | -35.65 * | -0.17 | 0.009 | -17.98 * |
| **S.length:Real** | -0.18 | 0.005 | -36.15 * | -0.06 | 0.005 | -13.46 * |

### Table S11

*ICM as an independent constraint: estimates from the fitted linear-mixed models for DL-matched random structures and DL-matched RLAs.*

| | DL-matched random structures | | | DL-matched RLAs | | |
|---|---|---|---|---|---|---|
| | **Estimate** | **Std. Error** | **t-value** | **Estimate** | **Std. Error** | **t-value** |
| **Intercept** | 1.15 | 0.005 | 243.39 * | 1.159 | 0.006 | 175.10 * |
| **S.length** | 0.11 | 0.004 | 27.24 * | 0.06 | 0.003 | 20.00 * |
| **Real** | -0.06 | 0.003 | -21.05 * | -0.02 | 0.003 | -7.43 * |
| **S.length:Real** | -0.04 | 0.002 | -19.39 * | -0.01 | 0.001 | -6.03 * |

### Table S12

*DLM as an independent constraint: estimates from the fitted linear-mixed models for IC-matched random structures and IC-matched RLAs.*

| | IC-matched random structures | | | IC-matched RLAs | | |
|---|---|---|---|---|---|---|
| | **Estimate** | **Std. Error** | **t-value** | **Estimate** | **Std. Error** | **t-value** |
| **Intercept** | 2.10 | 0.015 | 136.70 * | 1.86 | 0.015 | 121.56 * |
| **S.length** | 0.06 | 0.003 | 16.64 * | 0.26 | 0.007 | 34.85 * |
| **Real** | -0.02 | 0.016 | -1.53 n.s. | 0.001 | 0.008 | 0.10 n.s. |
| **S.length:Real** | -0.001 | 0.005 | -0.28 n.s. | -0.007 | 0.002 | -2.82 * |

## Note S14
## Languages used in the analysis

**Table S13**

*Names,ISO codes, Typology and Data Genres of the languages, from the SUD treebank, used for analysis.*

| Language | ISO | Typology | Data Genres |
|---|---|---|---|
| Afrikaans | af | SVO | legal, nonfiction |
| Amharic | am | SVO | bible, fiction, grammar-examples, news, nonfiction, |
| Arabic | ar | SVO | news, wiki |
| Armenian | hy | SVO | blog, fiction, grammar-examples, legal, news, nonfiction, |
| Bambara | bm | SVO | news, nonfiction |
| Basque | eu | SVO | news |
| Bulgarian | bg | SVO | fiction, legal, news |
| Catalan | ca | SVO | news |
| Chinese | zh | SVO | learner-essays, news, spoken, wiki |
| Croatian | hr | SVO | news, web, wiki |
| Czech | cs | SVO | fiction, legal, medical, news, nonfiction, reviews, wiki |
| Danish | da | SVO | fiction, news, nonfiction, spoken |
| Dutch | nl | SVO | news, wiki |
| English | en | SVO | academic, blog, email, fiction, government, grammar-examples, learner-essays, legal, news, nonfiction, reviews, social, spoken, web, wiki |
| Erzya | myv | SVO | fiction |
| Estonian | et | SVO | academic, blog, fiction, news, nonfiction, social, web |
| Faroese | fo | SVO | bible, fiction, nonfiction, wiki |
| Finnish | fi | SVO | blog, fiction, grammar-examples, legal, medical, news, poetry, social, web, wiki |
| French | fr | SVO | blog, legal, medical, news, nonfiction, reviews , spoken, wiki |
| Galician | gl | SVO | legal, medical, news, nonfiction |
| German | de | SVO | news, nonfiction, reviews, web, wiki |
| Greek | el | SVO | news, spoken, wiki |
| Hebrew | he | SVO | news |
| Hindi | hi | SVO | news, wiki |
| Hungarian | hu | SVO | news |
| Indonesian | id | SVO | blog, news, nonfiction, wiki |
| Irish | ga | SVO | fiction, government, legal, social, web |
| Italian | it | SVO | learner-essays, legal, news, nonfiction, social, wiki |
| Japanese | ja | SVO | blog, fiction, news, nonfiction, web, wiki |
| Kazakh | kk | SVO | fiction, news, wiki |
| Korean | ko | SVO | academic, blog, fiction, news, nonfiction, wiki |
| Latvian | lv | SVO | academic, fiction, legal, news, spoken |
| Lithuanian | lt | SVO | fiction, legal, news, nonfiction |
| Maltese | mt | SVO | fiction, legal, news, nonfiction, wiki |

**Table S13 continued from previous page**

| Language | ISO | Typology | Data Genres |
|---|---|---|---|
| Northern Kurdish | kmr | SVO | fiction, wiki |
| Northern Sami | sme | SVO | news, nonfiction |
| Norwegian | no | SVO | blog, news, nonfiction, spoken |
| Persian | fa | SVO | academic, blog, fiction, legal, medical, news, nonfiction, spoken, web |
| Polish | pl | SVO | fiction, news, nonfiction, social, spoken |
| Portuguese | pt | SVO | blog, news, wiki, |
| Romanian | ro | SVO | academic, bible, fiction, legal, medical, news, nonfiction, poetry, wiki |
| Russian | ru | SVO | blog, fiction, news, nonfiction, social, wiki |
| Serbian | sr | SVO | news |
| Slovak | sk | SVO | fiction, news, nonfiction |
| Slovenian | sl | SVO | fiction, news, nonfiction, spoken |
| Spanish | es | SVO | blog, news, reviews, wiki |
| Swedish | sv | SVO | fiction, news, nonfiction, spoken, wiki |
| Tamil | ta | SVO | news |
| Thai | th | SVO | news, wiki |
| Turkish | tr | SVO | grammar-examples, news, nonfiction, reviews, wiki |
| Uighur | ug | SVO | fiction |
| Ukrainian | uk | SVO | blog, email, fiction, grammar-examples, legal, news, reviews , social, web, wiki |
| Upper Sorbian | hsb | SVO | nonfiction, wiki |
| Urdu | ur | SVO | news |
| Vietnamese | vi | SVO | news |
| Wolof | wo | SVO | bible, wiki |

## References


Ferrer-i Cancho, R. F. (2004). Euclidean distance between syntactically linked words. *Physical Review E*, *70*(5), 056135.

Futrell, R., Mahowald, K., & Gibson, E. (2015). Large-scale evidence of dependency length minimization in 37 languages. *Proceedings of the National Academy of Sciences*, *112*(33), 10336–10341.

Gibson, E. (1998). Linguistic complexity: Locality of syntactic dependencies. *Cognition*, *68*(1), 1–76.

Prüfer, H. (1918). Neuer beweis eines satzes über permutationen. *Arch. Math. Phys*, *27*(1918), 742–744.

Straka, M., Hajic, J., Straková, J., & Hajic jr, J. (2015). Parsing universal dependency treebanks using neural networks and search-based oracle. In *International workshop on treebanks and linguistic theories (tlt14)* (pp. 208–220).

Yadav, H., Vaidya, A., Shukla, V., & Husain, S. (2020). Word order typology interacts with linguistic complexity: a cross-linguistic corpus study. *Cognitive Science*, *44*(4).
